# Modelling the potential of forest management to mitigate climate change in Eastern Canadian forests

**DOI:** 10.1038/s41598-023-41790-2

**Published:** 2023-09-04

**Authors:** Abderrahmane Ameray, Yves Bergeron, Xavier Cavard

**Affiliations:** 1 Institut de recherche sur les forêts, Université du Québec en Abitibi-Témiscamingue (UQAT), 445 Boul. de l’Université, Rouyn-Noranda, QC J9X 5E4 Canada; 2 Centre d’étude de la forêt, Université du Québec en Abitibi-Témiscamingue (UQAT), 445 Boul. de l’Université, Rouyn-Noranda, QC J9X 5E4 Canada

**Keywords:** Climate and Earth system modelling, Ecological modelling

## Abstract

Climate change poses a serious risk to sustainable forest management, particularly in boreal forests where natural disturbances have been projected to become more severe. In three Quebec boreal forest management units, biomass carbon storage under various climate change and management scenarios was projected over 300 years (2010–2310) with a process-based dynamic landscape model (PnET-succession for Landis-II). Several strategies varying in their use of partial cuts and clear cuts, including business as usual (BAU) (clear-cut applied on more than 95% of the managed area), were tested and compared to conservation scenarios (no-harvest). Based on simulation results at the landscape scale, the clearcut-based scenarios such as BAU could result in a decrease of biomass carbon stock by 10 tC ha^−1^ yr^−1^ compared to the natural scenario. However, this reduction in carbon stock could be offset in the long term through changes in composition, as clearcut systems promote the expansion of trembling aspen and white birch. In contrast, the use of strategies based on partial cuts on more than 75% or 50% of the managed area was closer to or better than the natural scenario and resulted in greater coniferous cover retention. These strategies seemed to be the best to maximize and stabilize biomass carbon storage and ensure wood supply under different climate change scenarios, yet they would require further access and appropriate infrastructure. Furthermore, these strategies could maintain species compositions and age structures similar to natural scenarios, and thus may consequently help achieve forest ecosystem-based management targets. This study presents promising strategies to guide sustainable forest management in Eastern Canada in the context of climate change.

## Introduction

Boreal forest ecosystems provide a wide range of ecosystem services. Forests offer a multitude of benefits beyond just providing wood products, as they also play an important role in filtering water supplies, controlling floods and erosion, sustaining biodiversity and genetic resources, and providing opportunities to mitigate climate change by reducing atmospheric CO_2_^1–4^. The boreal forests sequester 500 TgC yr^−1^ as a net carbon sink and store 25 and 60% of the world’s carbon biomass and soils, respectively. Furthermore, Smyth et al.^5^ point out that forest management and harvest of wood products may increase the mitigation potential of Canada’s forest sector by 68 and 320 TgC in 2030 and 2050, respectively. Forest management can improve carbon sequestration and storage capacity at the landscape scale^6^.

Climate change is increasing both frequency and severity of natural disturbances in Eastern boreal forests, which has an impact on all carbon pools and several processes such as decomposition, forest regeneration, and succession^7–9^. Wildfires, windthrows, and spruce budworm (SBW) outbreaks are the primary natural disturbances in Quebec, which play an important role in shaping forest age structure and composition^8,10,11^. In this part of the boreal biome, those disturbances could be classified according to the disturbed area, severity, and carbon impacts in the following order. First are wildfires, which are the most extensive disturbance, with the current annual burn rate varying between 0.04 and 0.26% in Quebec^8,12^. It is expected that this rate will increase under climate change, as indicated by studies^8,12^. Recent studies^12–14^ predict an increasing burned area trend in Quebec forests over the next 100 years, with inter-annual variability, mainly under RCP4.5 and RCP8.5 climate change pathways, thus increasing carbon losses from the biomass carbon pool^14^. Second is SBW outbreaks, which are characterized by a return interval of 32 years in the eastern regions of Quebec^15^. The last outbreaks was recorded in 1992 and decreased the average aboveground biomass and belowground biomass by 5.96 and 6.94%, respectively^16^. Dymond et al.^17^ state that SBW significantly reduced ecosystem carbon stock enough to change the landscape from a sink (4.6 ± 2.7 gC m^−2^ y^−1^ in 2018) to a source (− 16.8 ± 3.0 gC m^−2^ y^−1^ in 2018). Lastly, windthrows have a potential impact on the carbon cycle by increasing tree mortality^18^. The amount of damage caused by wind varies by tree size classes; while large trees may be relatively well anchored and fail via crown or stem break, smaller trees are frequently broken when large trees fall on them^19^. In addition, their effects are related to the species' root architecture and soil properties^19^. However, the effects of wind impacts are less than those of fires and SBW, as they affected, on average, 0.0255% of the area per year between 1971 and 2000^11^. All of the three cited disturbances cause carbon loss, increase tree mortality, and a greater amount of carbon being transferred from the live biomass pool to deadwood^18,20,21^.

The net primary productivity (NPP) and biomass carbon storage are directly influenced by local abiotic factors such as temperature, precipitation, atmospheric CO_2_ concentration, and solar radiation^14,22,23^. Since the late nineteenth century, the climate has been warming due to an increase in radiatively active gases in the atmosphere resulting from human activities. This warming trend is expected to have particularly pronounced impacts on higher latitudes, with anticipated increases in both precipitation and temperatures^24^. An increase in temperatures may stimulate vegetation growth up to a certain limit, beyond which it might reduce productivity under drought conditions or even cause increased mortality rates^25^. It is only in the last few years that it has been possible to assess the effect of climate change on the forest carbon cycle, using simulation models which couple plant-soil carbon with the nitrogen and water cycles, integrate atmosphere-vegetation interactions, and represent competitive processes^23,26–28^. Several models report that boreal forests are expected to accumulate more carbon in living biomass under global warming effects, mainly under intermediate scenarios (RCP2.6 and RCP4.5)^29,30^. However, climate (i.e., precipitation and temperatures) has many potential effects on forest growth, mortality, disturbances, and establishment^29^.

In addition to natural disturbances and local abiotic conditions, harvest has a high effect on the forest carbon dynamics^31,32^. This effect is related to applied canopy removal intensity (CRI). Indeed, 100% CRI using clear-cutting (CC) at the stand scale achieves negative net ecosystem production 20 years following harvest because of high respiration and a lower NPP of the replacing stand^6,31,32^. On the other hand, lower or moderate CRI using partial cuts (PC) and selective cutting maintains the uneven-aged forest system and thus may maximize forest carbon sequestration and storage compared to clear cuts in the long term^33,34^. Several studies have used a variety of methodologies (empirical data^35^ or simulation^29^) to examine the CRI on forest carbon from empirical data at the stand level and the majority of their findings reflect that PC with lower and/or moderate CRI could increase stand net growth and stabilize biomass carbon storage as well as improve the soil carbon sink^6^. In Quebec boreal forest, CC systems including CC and careful logging around advanced growth (CLAAG) (known also as Cutting with Protection of Regeneration and Soil (CPRS), which cuts around 95% of the canopy and aims to conserve advanced regeneration), and PC systems (such as shelterwood cutting systems, pre-commercial thinning, and commercial thinning with the protection of small merchantable stems) are applied^36^. Currently, in Quebec, more than 95% of the annual harvest area is managed using CC systems (CC and CPRS)^36^. However, their impacts at the landscape scale have not been well evaluated. Yet, forest planning and management decisions are always made at the regional (landscape) scale ^6^. Consequently, we must assess the impact of various harvesting intensities at the landscape scale, where different CRI such as PC and CC or CPRS could be used.

The annual harvest area percentage per treatment that ensures maximum carbon sequestration and storage, wood supply for industries, and conservation of current habitat (composition and age structure) is unknown. As a hypothesis, we expect that the inclusion of more PC systems at the landscape scale will improve biomass carbon storage since lower/moderate CRI could increase carbon sequestration by 36–40% in the boreal forest region^37^. In this paper, our main objectives were: (i) firstly, to project the effect of forest management and climate change on forest composition and age structure under climate change scenarios for 300 years (2010–2310). (ii) Secondly, to predict the accumulative impacts of SBW, wildfires, and windthrow on the biomass carbon pool (below and above ground) along with their interactions with climate change. (iii) Thirdly, to investigate the potential impact of forest management on carbon biomass storage, as well as annual harvested biomass. To achieve these objectives, a portfolio of strategies including variable proportions of low- and high CRI treatments (PC and CC), including the business-as-usual (BAU), were tested and compared to natural evolution (no-harvest) under different climate change scenarios. Using a mechanistic model (PnET succession for LANDIS-II), this study introduces a new methodology to evaluate the combined effects of forest management and climate change (through alterations of growth and mortality as well as of natural disturbances regimes) on carbon dynamics at the regional scale in the high latitudes of the eastern boreal forest of Canada.

## Materials and methods

### Study area

**Figure 1:**
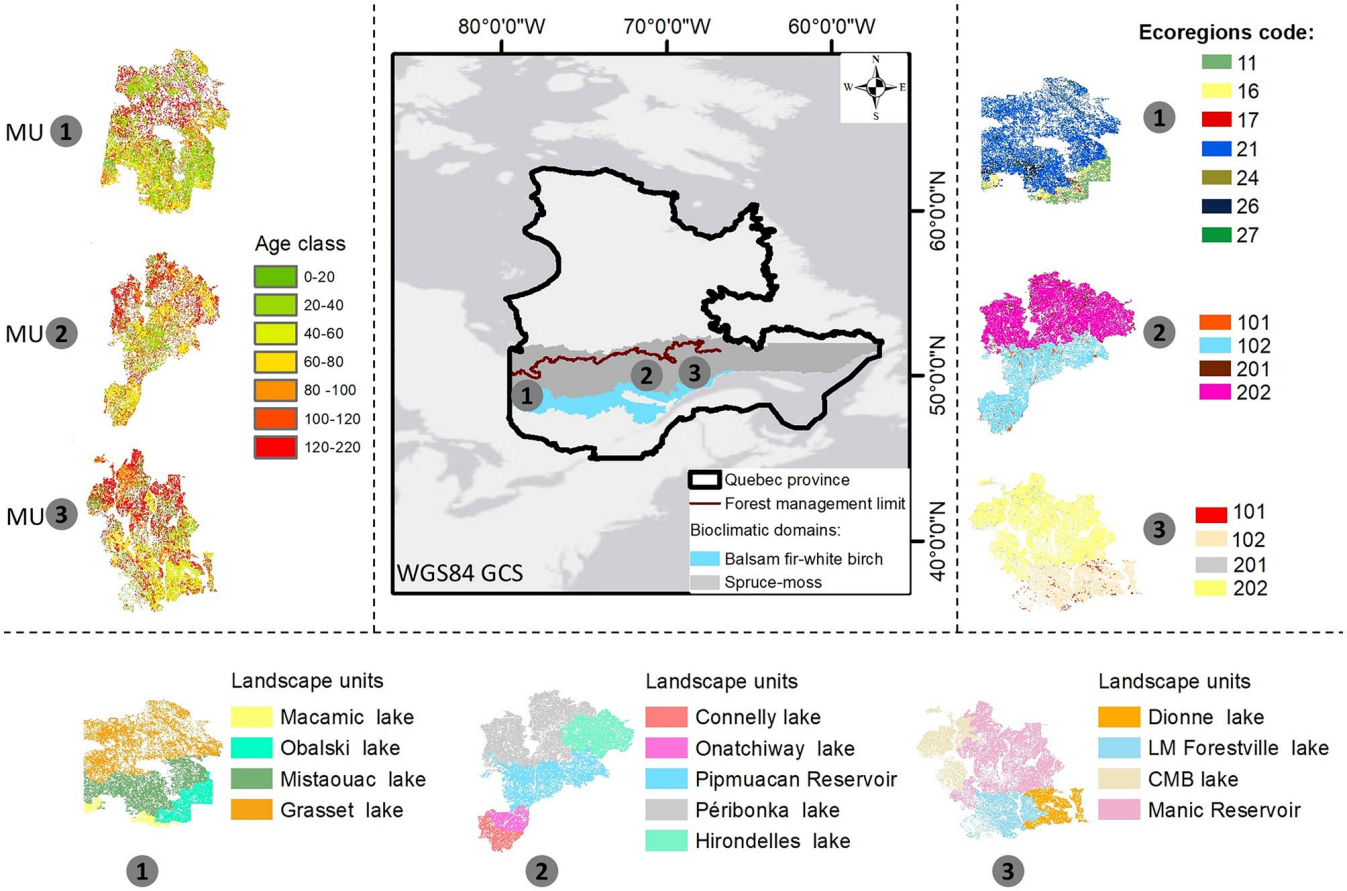
Study area (three MUs: 1, 2, and 3), with the forest age class structure at the beginning of the simulation (year 2010), as well as the landscape units inside each MU used as management areas. Each ecoregion per MU was characterized by a code map during the simulation, the soil texture of each one of them is in Table 1.

The study was conducted in three management units (MUs) near Quebec’s northern forest management limit, i.e., the limit of the territories where it was estimated that logging could be carried out profitably, and beyond this limit (~ latitudes > 51°), where forests are not managed because of their lower productivity^38^. These three MUs are distributed along a longitudinal gradient from west to east: North-du-Québec (MU1; UM08551), Saguenay-Lac-Saint-Jean (MU2; UM2471), and Côte-Nord regions (MU3; UM09351) (Fig. 1). These regions experience a boreal climate characterized by long, cold winters with short, mild summers and moderate, seasonally distributed precipitation^39^. Under current climate conditions, rainfall increases from east to west from about 800 mm to about 1000 mm, reflecting a longitudinal gradient between MU, while within each MU, a latitudinal temperature gradient that increased by around 2 °C from south to north was observed between ecoregions^39^. These MUs belong to the spruce-feathermoss and balsam fir-white birch bioclimatic domains of the Boreal Shield, dominated by black spruce (*Picea mariana*), jack pine (*Pinus banksiana*), balsam fir (*Abies balsamea*), white birch (*Betula papyrifera*), trembling aspen (*Populus tremuloides*) and other species^36^. The soils are characterized by a high clay content in MU1 and a high sand-silt percentage in MU2 and MU3.

### Climate data

The monthly climate data (temperatures, precipitation, radiation) for each ecoregion were extracted from the climateNA model and the second-generation Canadian Earth System Model (CanESM2) data was used^39^. Our analysis includes four climate scenarios: baseline (historic), RCP2.6, RCP4.5, and RCP8.5. The baseline climate scenario from 2010 to 2310 was constant around the monthly means of the historic climate (1900–2010). For RCP scenarios, the climateNA model provides data until 2100, so they were extrapolated at monthly intervals until 2310, using Extended Concentration Pathways (ECPs) rules from Meinshausen et al.^40^ and Collins et al.^41^, which describe the RCPs from 2100 to 2500 (see Ameray et al*.*^14^ for more details). CO_2_ concentration for RCP scenarios was acquired from the RCP database (https://tntcat.iiasa.ac.at/RcpDb), which covers data representing 10-years time frames until 2310. We assumed that CO_2_ remained constant at 389 ppm (2010) during the simulation for the baseline climatic scenario.

### Simulation models

#### Forest growth and succession model

**Figure 2:**
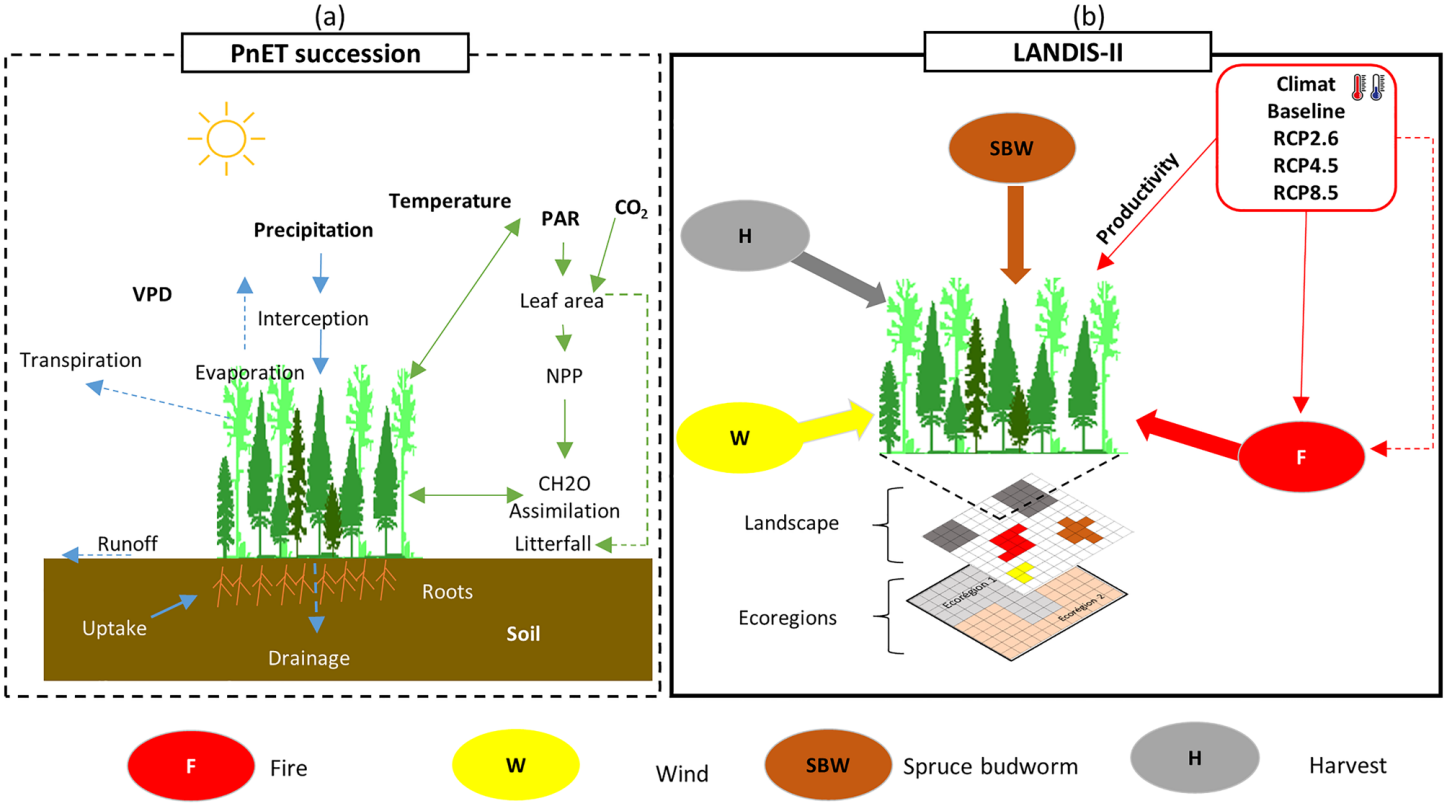
General methodology framework. (**a**) PnET model used for succession simulates simultaneously water and carbon cycles and integrates environmental factors such as soil texture, precipitation, temperature, radiation (PAR), and vapour-pressure deficit (VPD) to estimate the net primary productivity. (**b**) An overview of all the disturbance extensions from Landis-II considered in this study and their interaction at landscape scale per ecoregions.

LANDIS-II is a process-based, stochastic, and spatially explicit forest landscape model that integrates disturbance and succession models (Fig. 2b). The landscape in Landis-II is defined as a grid of cells, where each one of them is assumed to be homogeneous in terms of the stand, belongs to a homogenous ecoregion (soil and climate), and can contain one or multiple species cohorts that can be killed by age-related mortality, disturbances, and competition. In our study, we used PnET-Succession extension (V4.1), which embeds elements of the PnET ecophysiology model, and allows us to assess species establishment, growth, mortality, and competition for available light and water and links those processes to climate drivers^28,42,43^. The PnET extension is convenient for forest landscape modeling in a changing environment integrating precipitation, temperature, radiation, and atmospheric CO_2_ concentration (Fig. 2a)^28^. Furthermore, this model scales leaf-level processes such as respiration, transpiration, and photosynthesis with a monthly time step to the grid cell by incorporating light extinction and water consumption in stacked canopy layers and computing a dynamic soil water balance (Fig. 2a)^44^. PnET extension parameters such as foliar N concentration, maintenance respiration, half saturation light level for photosynthesis, and maximum and minimum temperatures for photosynthesis were calibrated and validated for the same MUs in previous works^14^, using Pothier and Savard empirical yield curves^45^. All simulations were run for 300 years (2010–2310) at 20-year step intervals and a 200 × 200 m resolution grid (4 ha). The simulated area was 0.62, 1.16 and 1.15 Mha in MU1, MU2, and MU3, respectively.

#### Initial landscape: species and ecoregions

Regarding the LANDIS-II inputs, life history traits of studied dominant tree species were retrieved from previous studies^46^, and included longevity, sexual maturity, shade tolerance, fire tolerance, seed dispersal distance, sprouting, and post-fire regeneration (supplementary material 1). For each MU, the initial forest composition and spatial distribution were derived from “Ministère des Ressources naturelles et des Forêts” (MRNF) geodatabase^36^ and rasterized to a fine spatial resolution (200 m), where each cell included the tree species and age-class information. At the beginning of the simulation (2010), the initial dominant age class was 20–40 years in MU1 and 120–200 years in both MU2 and MU3 (Fig. 1). The ecoregions were used from Ameray et al.^14^, where MRNF ecoregions were intersected with Duchesne and Ouimet’s^47^ soil map and MRNF’s bioclimate shapefile to have more refined ecoregions (Table 1). Each ecoregion was associated with a code map in raster format (Fig. 1). Water bodies, wetlands, islands, and other non-commercial species were excluded from our analysis (inactive cells).

**Table 1:** Simulated management units (MU) and their ecoregions.

Region	MU	Bioclimatic domains	ecoregion code	Soils texture	Area (%)
Quebec Nord-West	MU 1	Balsam fir-white birch	11	Clay	12
			16	Clay loam	06
			17	Loam	01
		Spruce–moss	21	Clay	72
			24	Sand clay loam	02
			26	Clay loam	06
			27	Loam	02
Saguenay lac-saint Jean	MU 2	Balsam fir-white birch	101	Sand	03
			102	Sandy loam	35
		Spruce–moss	201	Sand	04
			202	Sandy loam	57
Côte-Nord	MU 3	Balsam fir-white birch	101	Sand	02
			102	Sandy loam	30
		Spruce–moss	201	Sand	04
			202	Sandy loam	64

#### Natural disturbances

In order to model forest biomass carbon storage response to natural disturbances and different management scenarios under climate change (Fig. 2b), several extensions from the Landis-II library were used, including “Base Fire v4.0”^48^, “Base Wind v3.1”^49^, “Biomass Harvest v4.4”^50^ and “Biological Disturbance Agent (BDA) extension v4.0.1”^51^. Wildfires were included using the Base-Fire extension. This extension simulates fire regimes through stochastic fire events depending on ignition probability, fire size (min, mean and max), fire severity, and the *K* parameter that determines the strength of the association between fire spread probability and fuel age^48^. Fire regime input parameters were used from previous works in the Quebec boreal forest and calibrated for each climate scenario^3,8,12,52,53^. In addition, the Base Wind extension stochastically simulates windthrows disturbance based on their intensity, size, spread, severity, and rotation period^49^. The wind size and rotation per ecoregion were parameterized based on historical data from the forest inventory geodatabase^36^. Similarly, the BDA extension stochastically introduces periodic defoliation events uniquely parameterized by defoliator species; SBW disturbance^51^. Host tree species for SBW included, from the most to least vulnerable, balsam fir (*Abies balsamea*), white spruce (*Picea glauca*), and black spruce (*Picea mariana*). Parameters used in this study for BDA extension were calibrated and validated using various sources for the boreal forest^46,54^. We assumed that climate change did not directly affect the wind and SBW disturbance regimes, but rather altered forest composition and structure which influenced their spatial and temporal pattern.

#### Harvest disturbance

The biomass harvesting extension was used to model different management strategies^50^. This extension requires dividing the landscape into management areas (i.e., landscape units in Fig. 1), specifying stands to be harvested based on species and age criteria, and defining the order in which they will be harvested. We used the proposed annual harvested volume (converted to biomass) necessary to cover the timber supply from the 2023–2028 management plan (Supplementary material 2)^55^, corresponding to the current allowable annual cut (AAC), i.e., the maximum volume that can be harvested annually without reducing future forest production capacity, set by the Chief Forester of Quebec^55^. At the first-time step, all the scenarios were calibrated to cover the current AAC using the current climate (baseline). Firstly, we designed the prescriptions which reflected the silvicultural treatments used for harvest at stand scale^36^, including clear cut (100% CRI), CPRS (CRI was fixed at 95% and the cohorts of 1–20 years were conserved) and three forms of PC (with 25%, 50%, and 75% CRI). The stand ranking was based on species’ economic value and their minimum age criteria for exploitability. Secondly, for the BAU scenario, we used a geodatabase of the harvest history (1970–2010) to calculate the harvested managed area per silvicultural treatment (Supplementary material 3). Finally, we designed and compared other strategies to BAU, varying the harvested area per treatment (Table 2). In the Quebec boreal forest, where reforestation could follow harvest mainly after CC or CPRS, our prescriptions respected the historic planted ratio of each species (70, 25, 3, and 2% of black spruce, jack pine, larch tree, and white spruce, respectively). Furthermore, in our proposed scenarios the annual level of the replanted area was variable (Table 2).

**Table 2:** Tested scenarios and their description. Treatments used at stand scale with different harvesting intensities and the percentage of annually managed area per treatment. CC + reforestation is the percentage of the replanted area after harvest by CC or CPRS, due to a lower soil seed bank and regeneration rate.

Scenarios	Description	Code	Used treatment at stand scale and % of managed area per treatment					
			CC + regeneration	CC + reforestation	CPRS	PC75%	PC50%	PC25%
Scenario-0	No harvest scenario under natural disturbances	S0	0.0	0.0	0.0	0.0	0.0	0.0
Scenario-1	All the annually harvested area is managed using high CRI (CC and CPRS). The establishment is based only on regeneration	S1	50.0	0.0	50.0	0.0	0.0	0.0
Scenario-2	BAU. Currently used scenario, where CPRS and CC are used for more than 95% of annually harvested area and 10% is managed using PCs with 25%, 50%, and 75% of CRI	S2	From the historic, See Supplementary material 3					
Scenario-3	We used 75% of the annually harvested area for high CRI (CC and CPRS) and 25% for low-removal ones (PC)	S3	25.0	25.0	25.0	8.3	8.3	8.3
Scenario-4	We used 50% of the annually harvested area for high-removal treatments and 50% for low-removal ones	S4	16.7	16.7	16.7	16.7	16.7	16.7
Scenario-5	We used 25% of annually harvested area for high-removal treatments and 75% for low-removal ones	S5	8.3	8.3	8.3	25.0	25.0	25.0
Scenario-6	Extreme use of PCs (100% of annually harvested area), the opposite of scenario 1	S6	0.0	0.0	0.00	33.3	33.3	33.3

### Simulation settings

Simulations were carried out for 300 years, with three replicates to estimate uncertainties in the simulation results. We began our simulation with an only-succession scenario, where there are no disturbances. After we assessed the cumulative impact of natural disturbances, this was done by running the following scenarios: (1) only succession and winds; (2) only succession, winds, and fires; and (3) only succession, winds, fires, and SBW, corresponding to natural scenario (S0). Also, we aimed to compare CC based scenarios (S1, S2, S3) and PC based scenarios (S4, S5, S6) in the selected management units with natural evolution (S0) (CRI = 0 or total conservation) (Table 2). All the scenarios were designed to assess whether, and to what degree, different harvest practices, in combination with wind, wildfires, and SBW disturbances affect the changes in living biomass carbon pool and harvested biomass under climate change. In this study only living and harvested biomass were investigated. The total biomass harvested per management scenario was converted to merchantable proportion^4^ and compared to ACC. The differences in the biomass carbon pools between the management scenarios and the no‐harvest scenario (*Δ*_i,j_) were calculated in order to assess the biomass carbon balance of each management strategy (*S*_*i,j*_) relative to the natural scenario (*S*_*i,0*_) (Eq. (1))^56^.

1 $${\Delta }_{i,j}={S}_{i,j}-{S}_{i,0}$$

where *i* climate scenario: baseline (*i* = 1), RCP2.6 (*i* = 2), RCP4.5(*i* = 3), RCP8.5(*i* = 4), and *j* management scenario from 1 to 6 (Table 2).

The biomass community output extension was used to track each cell stand composition and the following forest type descriptions: BSPF: black spruce pure forests, OCPF: other coniferous pure forests (jack pine, balsam fir, white spruce, larch tree), BPF: broadleaves pure forests, BSJP: black spruce and jack pine, BSBF: black spruce and balsam fir, BSOC: black spruce and other coniferous, OCMF: other coniferous mixed forests, BMF: broadleaves mixed forests; BsBMF: black spruce and broadleaves mixed forests, and OCBMF: other coniferous and broadleaves mixed forests. To catch the effect of management on forest composition, the differences between each composition per management scenario and its correspondence in the natural scenario (no harvest) were calculated. All analyses were carried out in Python environment.

## Results

### Forest management effect on composition and age structure

**Figure 3:**
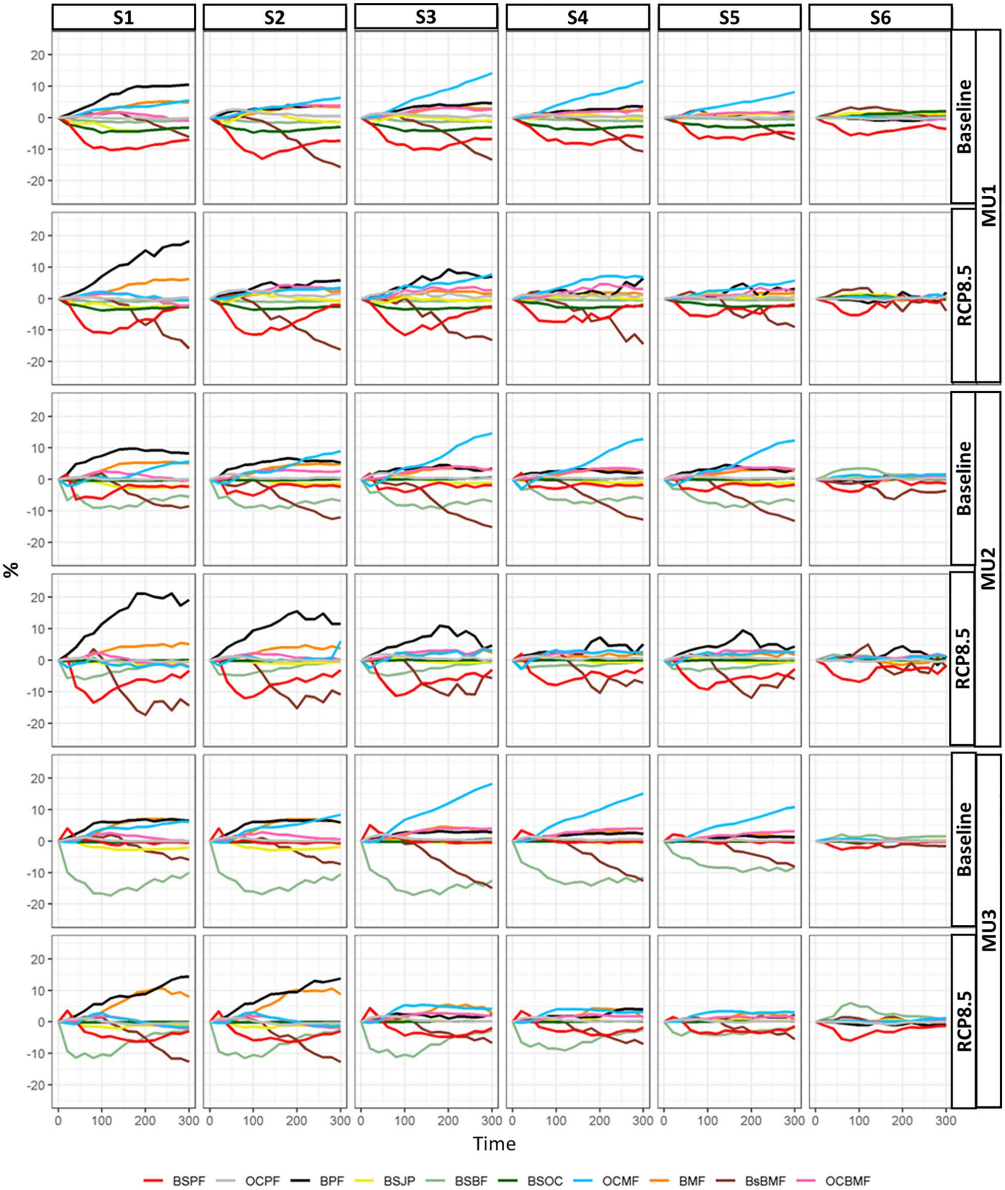
The difference (*Δ*; %) of composition percentage between natural scenario and management scenarios in the three MUs between 2010 (year 0) and 2310 (year 300) under current climate scenarios and RCP8.5. (BSPF: black spruce pure forests, OCPF: other coniferous pure forests, BPF: broadleaved pure forests, BSJP: black spruce and jack pine, BSBF: black spruce and balsam fir, BSOC: black spruce and other coniferous, OCMF: other coniferous mixed forests, BMF: broadleaved mixed forests; BsBMF: black spruce and broadleaved mixed forests, OCBMF: other coniferous and broadleaved mixed forests). The difference results under RCP2.6 and RCP4.5 are in supplementary materials 4.

According to our simulations at the landscape scale in all management units (MUs), the following compositions show the most significant alterations due to management and climate change when compared to natural evolution (S0): BSPF, BSOC, BSBF, BsBMF, OCMF, BPF, and BMF (Fig. 3, Supplementary material 4). In MU1, under the current climate, the differences between all scenarios and S0 showed that the strategies based on CC (S1 and S2 (BAU), and S3) may reduce BSPF, BSOC, BSBF, and BsBMF by 10, 5, 2, and 15%, and generally increased BPF, BMF, and OCMF by 10, 5 and 13%, respectively. In MU1, BPF may rise by 20% under RCP8.5 and S1 because this scenario was based on regeneration rather than the reforestation of coniferous species. In MU2, CC-based strategies (S1, S2 and S3) reduce BSPF, BSBF, and BsBMF by more than 8, 10 and 15%, respectively, independently of climate scenarios (Fig. 3, Supplementary materials 4). In contrast, in this unit BPF may attain more than 10, 7 and 5% under S1, S2 and S3, respectively; similarly, BMF had increased to more than 5% for all CC-based scenarios. These BPF thresholds may double in MU2 as a result of climate change (Fig. 3, Supplementary Material 5), in particular for RCP8.5. The most altered composition in MU3 under the current climate is BSBF, which decreased by 18, 17, and 18% under S1, S2, and S3, respectively, compared to S0. These three scenarios in MU3 may decrease BSBF, BSPF, and BsBMF by 10, 5, and 15% under RCP8.5. Briefly, CC-based strategies increased the abundance of broadleaved species and decreased coniferous cover in all MUs, primarily black spruce, regardless of climate scenarios.

On the other hand, PC inclusion (S4, S5 and S6) diminished the gap between management and natural evolution (S0) (Fig. 3, Supplementary materials 4). Generally, the differences of all compositions when compared to S0 under S6 scenarios were less than 5% in all MUs under all climate change scenarios. For instance, in MU1 under the current climate, S4, S5 and S6 had decreased BSPF by 8, 7, 4, and BsBMF by 15, 9 and 1%, respectively. Similarly, under RCP8.5 in MU1, BSPF dropped by 4, 3 and 2% under S4, S5, and S6 respectively; in MU2, for the baseline climate scenario, BSBF decreased by 8% under S4 and S5, but increased by 2% under S6. BSPF decreased by 2% under S4 and S5, but only 1% was observed under S6. Additionally, BsBMF differences were predicted to be less than 12% for the S4 and S5 scenarios and less than 5% for the S6 scenario; in MU3, BSPF composition under the current climate was stable (~ 0% of the difference compared to S0) under PC-based strategies (S4, S5 and S6). BSBF dropped by 12% and 10% under S4 and S5, respectively, and increased by 1% for S6 under the current climate.

**Figure 4:**
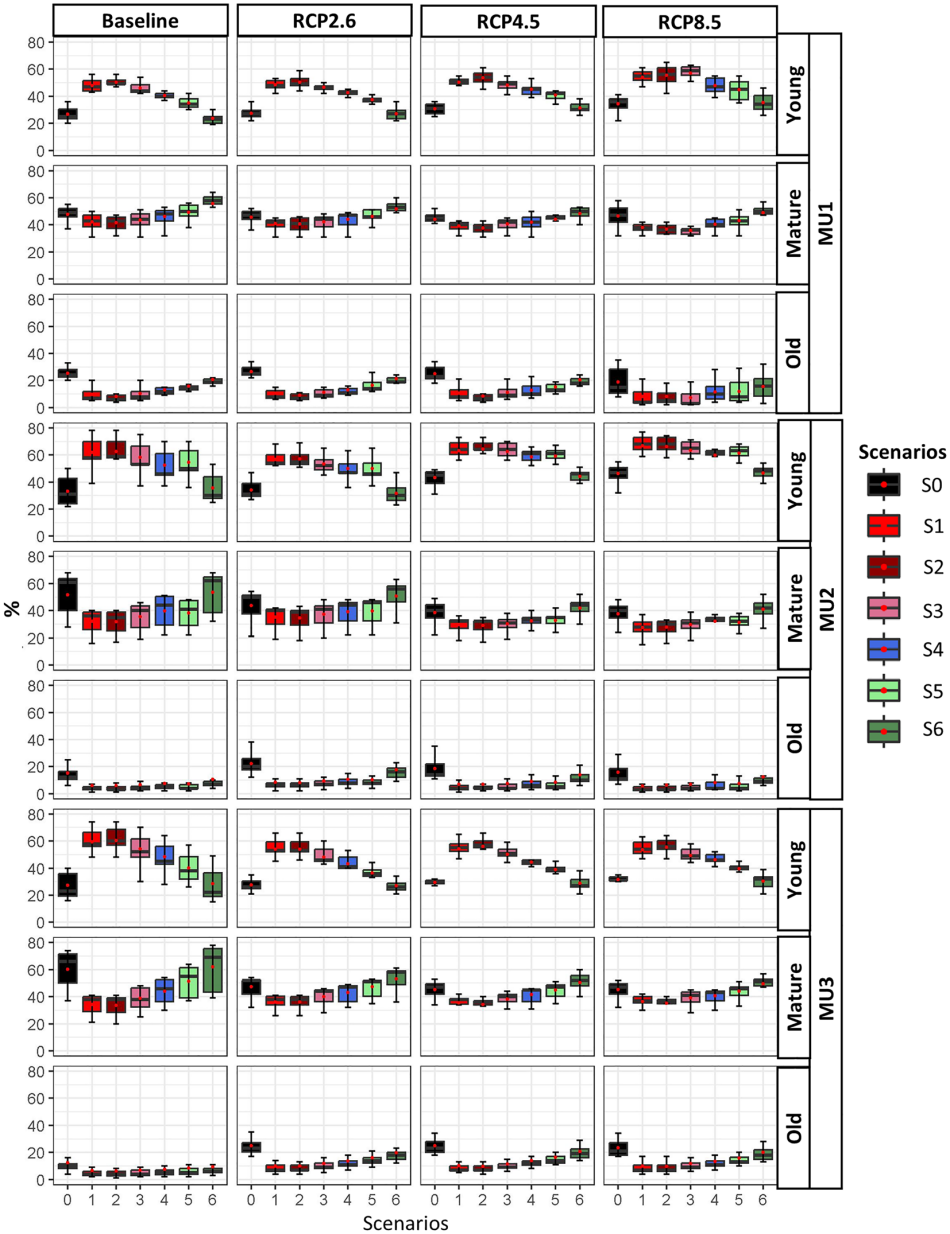
Age structure average of young forest (age ≤ 40), mature forest (40 < age ≤ 100), and old growth forest (age > 100) during the entire period under climate change and all management scenarios.

Regarding the age structure, independently of climate scenario in all MUs, the abundance of young forests in S1 and S2 (BAU) increased by more than + 25% and mature and old-growth forests decreased by -10% and -16%, respectively, compared to the natural scenario (S0) (Fig. 4, Supplementary materials 6). The inclusion of PC could help to reverse the decline in mature and old-growth forests in all MUs. For instance, in MU1, the differences in the young forest from S0 were approximately + 20, + 17, + 15, + 10%, and − 2% under S3, S4, S5, and S6, respectively, whereas those differences were approximately − 5, − 3, − 2, 0, + 8% for mature forests in the same order. Additionally, according to our simulation, old-growth forests under S3, S4, S5, and S6 varied by − 14, − 10, − 7, and − 4%, respectively. MU2 and MU3 showed a similar age-structure pattern under various management scenarios. In conclusion, the application of PC-based strategies (lower and moderate CRI) on more than 75% or 50% of the managed area (S4, S5 and S6) mimicked the natural scenario by emulating similar natural vegetation patterns and age structures (Figs. 3, 4) under all climate scenarios.

### Cumulative impact of natural disturbances

**Figure 5:**
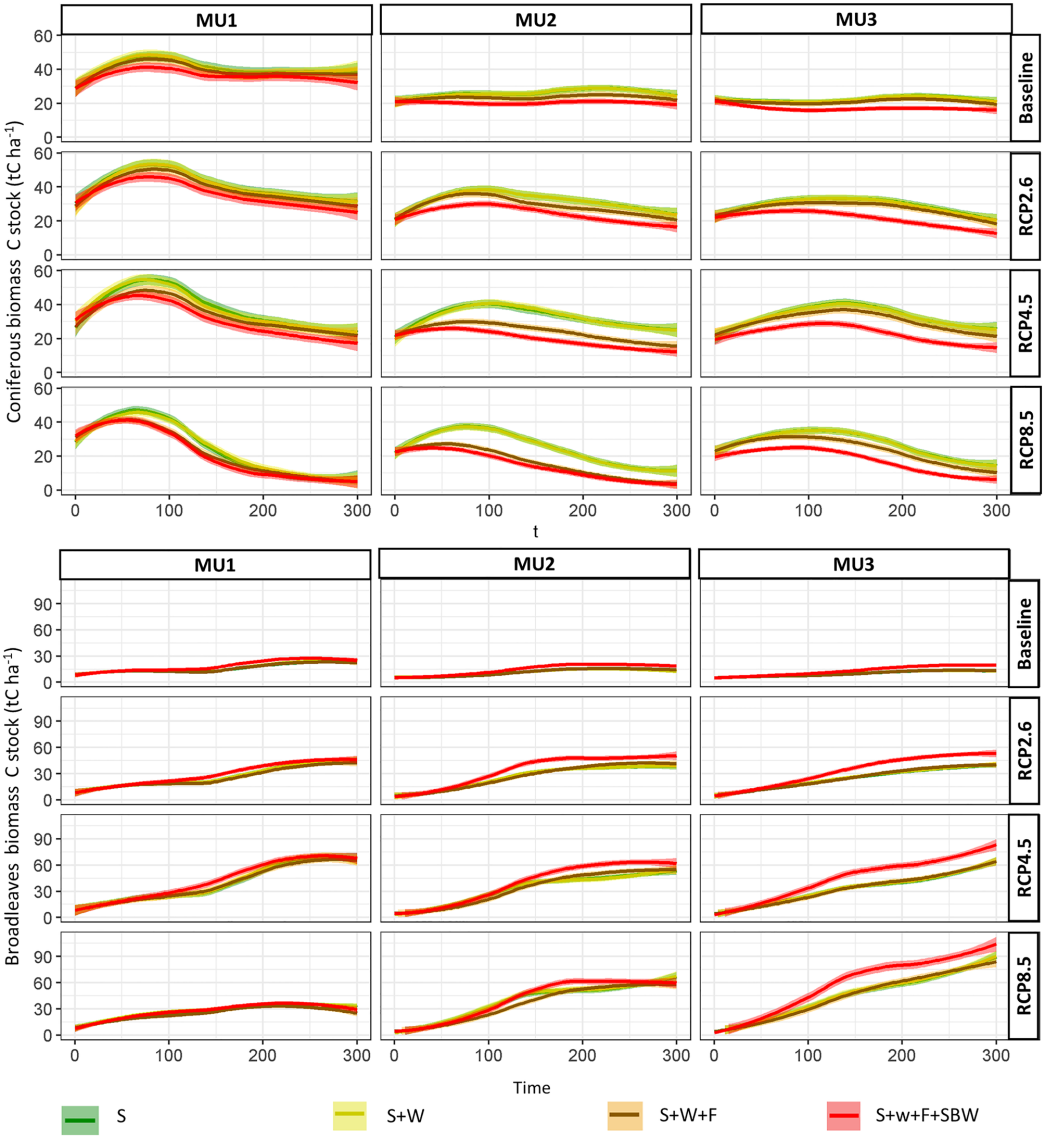
Accumulative impact of natural disturbances (legend: S = only-succession, W = winds, F = fire, SBW = spruce budworm) by 20-time step on biomass carbon storage for both coniferous and broadleaved species in the management unit under four climate scenarios (baseline and three RCP scenarios) during the study period 2010 (year 0) and 2310 (year 300).

Under the only succession scenario (no disturbances) and current climate (baseline), coniferous biomass carbon stock increased in MU1 from 27 to 37 tC ha^−1^ and remained stable in both MU2 and MU3 at around 26 tC ha^−1^ (Fig. 5). However, under RCP8.5 their biomass decreased by 72 and 25% in MU1 and MU2, respectively, in the simulation when compared to the baseline. Meanwhile, broadleaved species biomass carbon stock increased by around 200% in all MUs under RCP2.6 and RCP4.5 and could reach 300% in MU3 under RCP8.5 at the end of the simulation. Windthrow (S + W scenario) had a lower effect compared to the other disturbances (fire and SBW) (Fig. 5, Supplementary material 7). For instance, under the current climate, the average coniferous carbon biomass losses were − 0.03, − 0.12 and − 0.13 tC ha^−1^ yr^−1^ during the study period in MU1, MU2 and MU3, respectively. Fires (S + W + F scenario) had a considerable impact mainly on the coniferous biomass of both MU1 and MU2, due to shorter fire cycles. The biomass carbon storage of broadleaved species was close to that of the succession-only scenario for S + W and S + W + F scenarios. When SBW was considered (S + W + F + SBW scenario), the biomass carbon stock of coniferous considerably dropped mainly in MU3 compared to other scenarios and an increase in that of broadleaved species was noticed (Fig. 5, supplementary materials 7). For example, in MU3 where SBW is more frequent, the average annual losses of coniferous biomass carbon storage due to SBW were − 3.89, − 5.77, − 7.30, and − 6.26 tC ha^−1^ yr^−1^ for the baseline RCP2.6, RCP4.5, and RCP8.5, respectively (supplementary materials 7). On the other hand, the biomass carbon storage of broadleaved species was increased by 3.38, 8.27, 12.02, and 13.24 tC ha^−1^ yr^−1^ for baseline, RCP2.6, RCP4.5, and RCP8.5 respectively (supplementary materials 7). For the entire study period, the average reduction rate in biomass carbon storage for coniferous species vulnerable to SBW was 5.25, 19, and 23% in MU1, MU2 and MU3, respectively.

### Management effect on forest carbon

**Figure 6:**
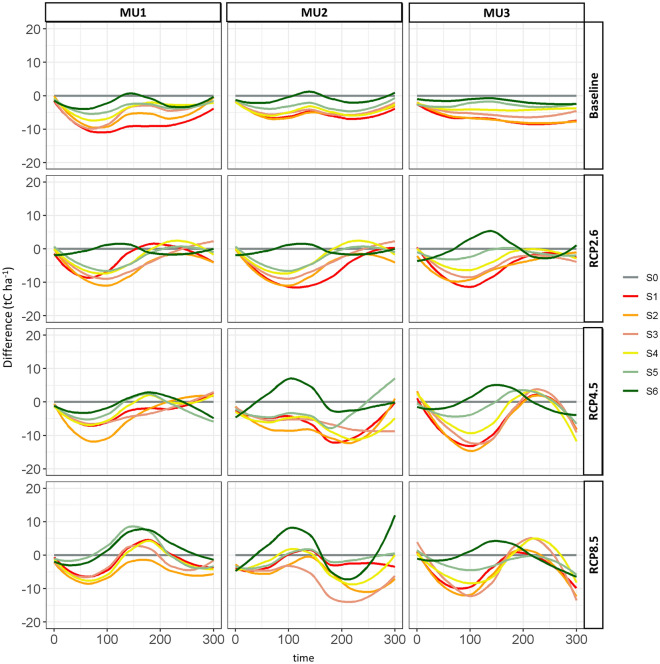
The living biomass carbon storage differences (*Δ*_i,j_) from 2010 (year 0) to 2310 (year 300) between management scenarios described in Table 2 and no harvest scenario (S0: natural disturbances only). The relative changes compared to the natural scenario (S0) expressed in percentages and the confident interval of *Δ*_i,j_ are in supplementary materials 8 (a) and (b) respectively.

CC-based scenarios (S1, S2 and S3) decreased the total biomass carbon storage by around to 10 tC ha^−1^ yr^−1^ over the next 100 years compared to the no harvest scenario (Fig. 6) under the current climate (baseline) in all MUs. On the other hand, PC-based scenarios (S4, S5 and S6) showed a better performance than CC-based scenarios, particularly for S5 and S6, which were close to natural scenarios (S0) with a difference less than 2 tC ha^−1^ yr^−1^ in all MUs under baseline climate scenarios. Under climate change, the losses under S1 and S2 attained 12 tC ha^−1^ yr^−1^ for MU1 and 15 tC ha^−1^ yr^−1^ in both MU2 and MU3. Nevertheless, those reductions in biomass carbon stock under strategies based on high CRI (i.e., S1, S2) could be compensated after 200 years (2210), because these scenarios accelerated the abundance of intolerant shade species (trembling aspen and white birch) compared to natural scenarios (Supplementary materials 5, Fig. 3), which captured more carbon and offset previous years’ losses. The use of PCs on more than 50% (S4), or 75% (S5) or 100% (S6) of the managed area was the best strategy to stabilize biomass carbon storage under current climate and may achieve high stocks under RCP scenarios compared to S0 in the next century (Fig. 6).

Regarding the reforestation effect, S3 with a 25% replanting rate after CC had lower biomass carbon storage than S4 and S5 which were based on 16.7 and 8.3% replanting rates, respectively, under all climate scenarios (Fig. 6). For example, under the current climate, S3, S4 and S5 reduced the biomass carbon storage by 9, 6, and 5 tC ha^−1^ yr^−1^, respectively, in MU1. Similarly in the same order, those carbon stocks dropped by 7, 7, and 4 tC ha^−1^ yr^−1^ in MU2. Also, the difference in carbon stocks between these three scenarios was considerable in MU3, where a reduction of 6, 4, and 2 tC ha^−1^ yr^−1^ was found for S3, S4 and S5, respectively. However, in all MUs, in terms of biomass carbon storage, S4 and S5 showed a higher performance than BAU (S2), while the effect of S3 was almost similar to BAU.

### Harvested biomass carbon storage

**Figure 7:**
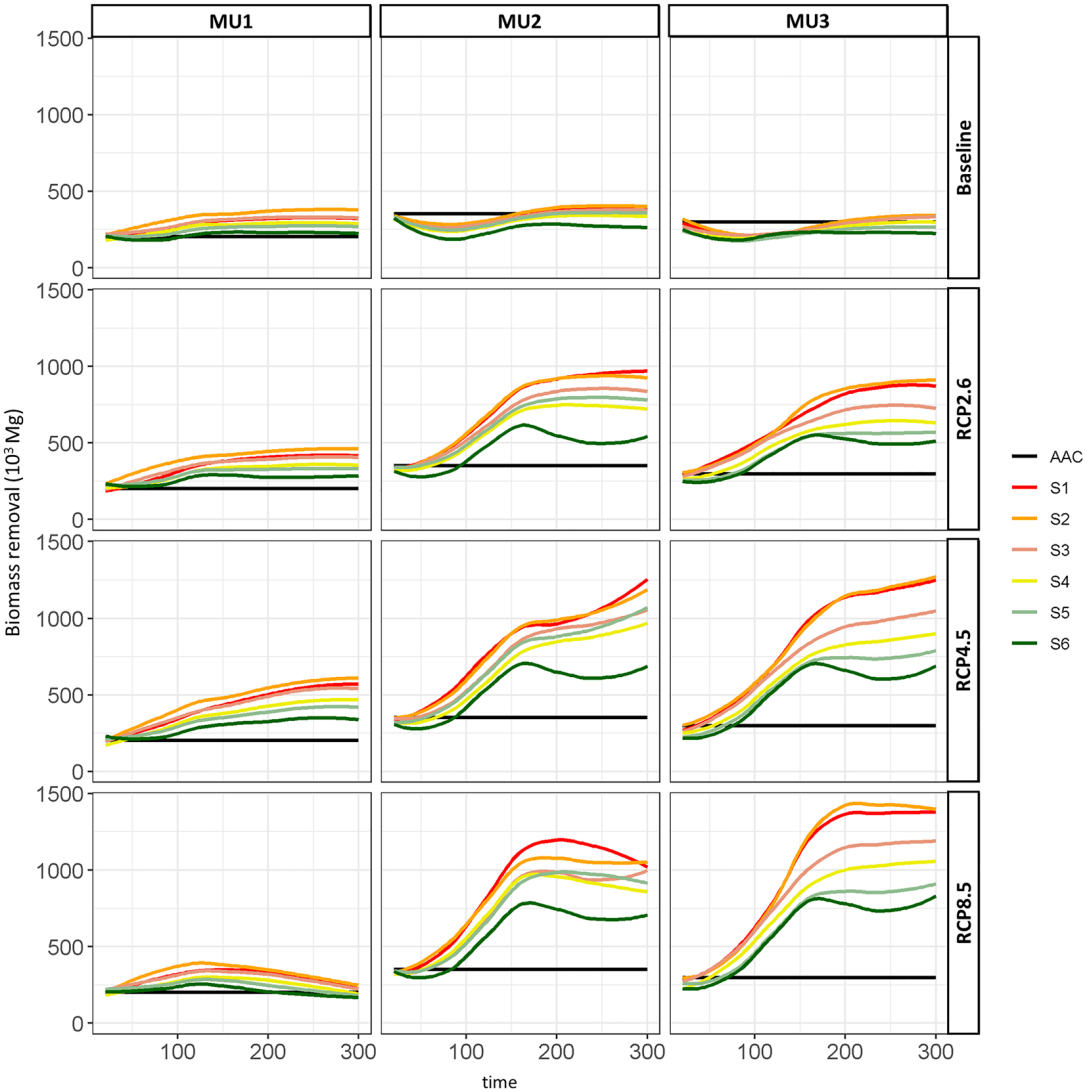
Annual harvested biomass (10^3^ Mg) per management scenario under climate change effect, compared to the allowable annual Cut (AAC of 2023–2028) from 2010 (year 0) to 2310 (year 300). All the scenarios were calibrated at the beginning of simulations to cover the timber supply analysis for the period 2023–2028 (AAC).

The estimated AAC of 2023–2028 was around 2.3 10^5^, 3.5 10^5^, and 4.0 10^5^ Mg yr^−1^ in MU1, MU2, and MU3, respectively (Fig. 7). Under baseline climate scenarios for all MUs, all the tested management strategies satisfied the AAC of 2023–2028 except S6 where the total landscape was managed using only PC (Fig. 7). In the short-term (2010–2110), the coniferous species represented more than 50% of annual harvested biomass, with mainly black spruce and jack pine in MU1, and black spruce with balsam fir in MU2 and MU3. After 110 years, more broadleaved species were harvested than coniferous (supplementary materials 9). Also under climate change, broadleaved species were the most harvested species in the medium and long term (period 110–310). Strategies with more PCs were able to keep greater coniferous contributions in AAC under the climate change effect (supplementary materials 9) since they maintained their cover retention over the medium and long terms (Fig. 3). The annual harvested biomass was expected to increase in MU1 and decrease in the next 100 years for both MU2 and MU3 under the current climate. However, under RCP scenarios the annual harvested biomass increased for all MUs, and all the strategies fulfilled the industrial demand. For instance, under RCP2.6 the annual harvested biomass could reach 5 10^5^, 10^6^, and 9 10^5^ Mg yr^−1^ for MU1, MU2 and MU3, respectively. Under RCP4.5 those amounts could reach 7 10^5^ Mg yr^−1^ in MU1 and more than 7 10^5^ Mg yr^−1^ in MU2 and MU3. Nevertheless, the annual harvested biomass dropped considerably under RCP8.5 in MU1 after 200 years.

**Figure 8:**
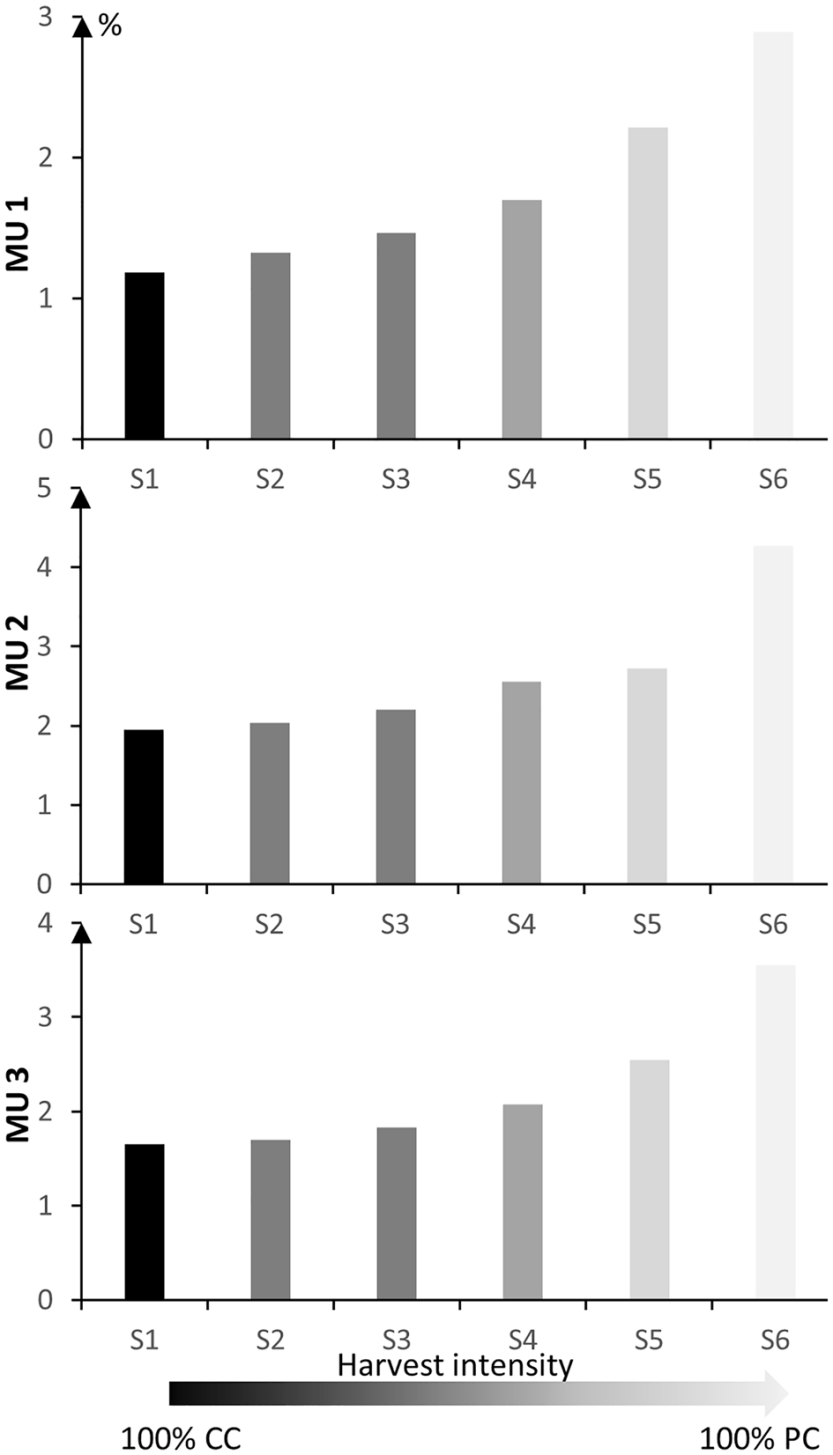
Annual harvested area (expressed in %) per management scenario. The threshold of S2 corresponds to the required annual managed area (%) to fulfill the timber supply analysis for the period 2023–2028 (AAC) under BAU scenario.

In order to fulfill the current AAC of 2023–2028, the harvested area using the BAU scenario (S2) was 1.32, 2.04, and 1.80% of the annual managed area in MU1, MU2, and MU3, respectively (Fig. 8). When PCs were used more than CC and CPRS, the harvested area to cover current AAC increased and doubled that of S1 and S2. For instance, under the S6 strategy it reached 2.89% yr^−1^ in MU1, 4.27% yr^−1^ in MU2, and 3.55% yr^−1^ in MU3. Nevertheless, because the annual harvested biomass is projected to rise as a result of climate change (Fig. 7), it is likely that the harvested area needed to fulfill the current AAC using S6 will be less than that indicated for the baseline climate.

## Discussion

### Climate change and natural disturbances impacts on biomass carbon storage

Evaluating the net effects of climate change on regional carbon dynamics requires the use of forest landscape models, such as the one we present in this manuscript, which includes the main driving processes and their interactions with local conditions (soil and climate). The results of this study are in agreement with previous studies^14,52,57^, which report that climate change will increase forest productivity in the high latitude of boreal forests. Other studies underline an increase in drought and mortality risk, mainly under RCP8.5 in the drier, western regions^14^. According to our projections, moderate climate change scenarios (RCP2.6 and RCP4.5) will likely increase and probably double the current productivity because of the shift in forest composition toward broadleaved species and changes in the capacity of trees to grow and sequester atmospheric carbon for both coniferous and broadleaved species^14^. This positive effect could be explained by the extension of the growing season and the reduction of potential cold-temperature late-frosts^57^. Our simulated short-term (2010–2110) responses in coniferous biomass carbon showed an increase in all MUs near the Quebec Forest management limit, although a reduction after 2110 was predicted in the western region. These findings are consistent with other empirical studies and model projections (*e.g*.,^58,59^), which concluded that increases in surface air temperature could hinder the capacity of coniferous species to grow and assimilate atmospheric carbon^57^. In addition, the increase in mixed forest occupancy, mostly between black spruce and balsam fir, black spruce and broadleaved species, and broadleaved pioneer forests of birch or aspen, might improve biomass carbon stock^6,60–62^. In the long term, there are a few studies that project carbon dynamics for time periods greater than 100 years in the boreal forest zone^63–65^. Our simulations suggest either a long-term decline or a stable trend over time of coniferous biomass stock in eastern and western regions, respectively, while that of broadleaved species will increase for all RCP scenarios. Likewise, boreal conifer biomass declines are projected to be more significant with increasing anthropogenic climate forcing and with decreasing latitude^64^.

Regarding composition, all of our results are consistent with previous projections in Quebec’s boreal forests^13,14,52,64^, which state that the future boreal forest composition will feature less pure black spruce forests and higher occurrences of (a) black spruce mixed with white birch and trembling aspen, (b) mixed forest of broadleaved species, (c) mixed forest between black spruce and other coniferous mainly jack pine and balsam fir. Our findings suggest that climate change will favor increased abundances of white birch and trembling aspen^64,66^. In contrast, extreme climate scenarios (RCP8.5) will possibly result in a more significant decline in coniferous communities compared to RCP2.6 and RCP4.5 after 2100 in the western regions of Quebec’s boreal forest^14^. Similarly, Boulanger et al.^64^ found that pioneer species proportions in the boreal forest could increase by 10–50% relative to the baseline climate scenario. In addition, natural disturbances could alter forest composition. For instance, Molina et al.^52^ pointed out that an increase in fire events is accompanied by an increase in mixed broadleaved species stand proportions in the landscape for western regions. Additionally, in MU3 under the current climate and the no-harvest scenario, compared to Ameray et al.^14^ where SBW was not considered, black spruce and balsam fir occupancy was around 30% in the medium and long term, but in this study, this composition was reduced to 21% because of the effect SBW (supplementary materials 5).

Carbon dynamics in North American boreal forest ecosystems are strongly affected by tree mortality that occurs during SBW outbreaks, fire events and windthrows. Our simulations show an increase in broadleaved species biomass carbon storage under SBW outbreaks. In fact, SBW killed the host tree species, i.e., balsam fir, white spruce, and black spruce, and increased empty cells which regenerated later with broadleaved species, thus explaining these increases. Similar to the findings of Liu et al.^16^, SBW defoliation and related mortality decreased the average coniferous biomass carbon stock by around 5–6% under the current climate. SBW outbreaks typically result in an average 42–50% stand mortality and a loss of biomass production of 32–48%^10,67^, with a value of 20–30% being found for the eastern units. Our findings are consistent with previous studies which report that an increase in wildfire activity could explain the declines in biomass^52,64^. The carbon losses from the biomass pool under fire events may reach 10 tC yr^−1^ in MU1 and MU2 by 2100 under RCP8.5 (supplementary materials 7)^14^, which could be explained by the increase in annual burn rate that may reach more than 1 and 1.25% in MU1 and MU2, respectively^8^. Fire cycles are generally shorter in the western regions, resulting in a younger post-fire forest with patches of older forest dispersed throughout with lower biomass carbon stock^64,68^. Under the current and extreme climate change scenario (RCP8.5), Splawinski et al.^69^ predict that over the next 50 years, the amount of forest area affected by natural regeneration failure will gradually increase because of increasing fire intensity, meaning the open woodland areas in our study might be underestimated, primarily in MU1 and MU2.

Windthrows are likely to further reduce carbon sink strength in specific regions of the boreal zone, but are not well quantified in previous works. In our simulations, windthrows were a minor driver compared with other natural disturbances and had a minor effect on biomass carbon dynamics compared to wildfires and SBW. Windthrow impacts in Canada’s boreal forest appear to be limited to occasional local events^11,70^. We emphasize that there was a high synergy between forest species to offset carbon losses under natural disturbances, as broadleaved species could offset the carbon losses from coniferous species (supplementary materials 7). In fact, a reduction in coniferous productivity is synchronized with an increase in that of broadleaved pioneer forests after disturbance^71^. The occurrence of birch (or aspen) as pioneer trees after disturbance is widely observed in the boreal forest biome^52,54,62^.

### Future management strategies

The impact of PCs has been identified as one of the major knowledge gaps in regional and global carbon accounting. In the last decade, PCs have started gaining interest as alternatives to CC to mitigate climate change and several studies (i.e.,^6,29,65^) show that they could enhance carbon sequestration rates. Based on our results at stand scale (1 cell of 4 ha), PC-based scenarios (S4, S5, S6) were close to natural scenarios and could stabilize biomass carbon storage in the long term (Supplementary materials 10). This effect could be explained mainly by the reduction in competition between cohorts for light and water since PC opens the canopy and increases available light and water for growth. Furthermore, PCs could reduce regeneration failure by conserving seed-trees and decreasing biotic and abiotic stresses through microhabitat modification under the tree canopy, resulting in a higher rate of germination and greater seedling survival^72^. Therefore, at the landscape scale the PC based scenarios could be favoured with respect to those based on CC systems^29,33,52,64^. Similarly, Lee et al.^33^ and Taylor et al.^29^ affirm that the positive effect of PC on forest carbon sequestration depends upon CRI. In addition, Simard et al.^73^ found that the high intensity 1-year post-harvest decrease in total biomass carbon stocks and the magnitude of these losses were negatively correlated with climatic aridity. From a long-term study, Peng et al.^65^ concluded that PC could increase carbon sequestration by about 36–40% in the boreal forest region. On the other hand, a few recent studies showed that there is a high interaction between PCs forms (*e.g.,* shelterwood cutting, selection (distant or close), retention systems, and seed-tree systems) and increased risks of disturbances, mainly windthrow^74^. Montoro Girona et al.^74^ state that 60% of residual trees were dead in seed-tree treatments, compared to 30% for shelterwood cuts. Therefore, the success of the PC approach requires consideration of not only its intensity but also its form^6^. Our study did not consider such interactions between PCs and windthrow as Landis-II is not spatially explicit within a cell.

In the eastern boreal forests of Canada, transient changes could be largely tackled through management interventions, such as changes in harvesting intensity and using more PCs at the landscape scale than CC. Our analyses showed that the BAU scenario will strongly decrease forest inertia and will interact with anthropogenic climate forcing to further alter forest landscapes in all MUs. Furthermore, strategies based on more CC and CPRS could accelerate the abundance of pioneer species and decrease coniferous communities at the landscape scale, which could explain the accelerated increase in broadleaved species biomass and the decrease in that of coniferous species for the scenarios which used more of those treatments. This study provides two promising strategies (S4 and S5) with different harvesting intensities, which could be the best direction for increasing forest carbon sequestration capacity and maintaining other ecosystem services^6^. These two strategies can maximize forest carbon biomass at the landscape scale and fulfill industrial needs, which ensures a positive trade-off between carbon sequestration and harvested wood products.

Reforestation is used in Quebec boreal forests mainly after CC and CPRS where regeneration is insufficient^75,76^. Our study shows that strategies with high reforestation rates may not achieve greater carbon sequestration than the natural scenario where carbon sequestered in harvested wood products is not taken into account, *e.g.,* strategy S3 where the rate was 25% of the annually managed area. It must also be considered that CC based scenarios (S1, S2, S3) harvest less area in addition to the high carbon transfer to harvested wood products (Fig. 7), compared to other PC based strategies. Furthermore, we emphasize that strategies with high reforestation rates, such as S3 (25% on annual harvest area), cannot achieve as high a cumulative photosynthesis value compared to the natural scenario, and their effect is almost similar to the BAU under different climate change scenarios. In terms of forest carbon stabilization at the ecosystem scale, our findings showed that scenarios S4 and S5 seemed to be the best ones in MU1 and MU2 under the current climate. The annual reforestation for the BAU is 15 and 10% in MU1 and MU2, respectively, which means that currently we are close to the S4 and S5 reforestation rates in these units (Supplementary materials 3). Therefore, S4 and S5 could be the best ones for MU1 and MU2. In the MU3 region, the increase in the reforestation rate is recommended to be at least 8.33%, which corresponds to the S5 strategy. Still, using S4 in MU1 with a 16.7% coniferous reforestation could show similar results to the BAU under RCP8.5, because their net primary productivity and biomass stocks were projected to be lower under extreme climate scenarios in this region^14^.

Forests are currently managed for multiple goals and benefits and not only to improve carbon sequestration^77^, and, because climate change is a dynamic and complex phenomenon with high uncertainty, any future strategy should at least maintain similar vegetation patterns and age structures to the natural scenarios under different climate change scenarios. With increasing pressures from global changes, sustainable forest management can probably be accomplished with an efficient combination of PCs, CC, and CPRS. Increasing the share of protected forest areas for biodiversity conservation is necessary, but has proven to be difficult, given mounting land-use pressures. In these circumstances, promoting strategies such as S4 and S5 that could maintain the old-growth forest, uneven-aged forest structures, and deadwood (enriching soil organic carbon) may be required to halt further degradation of biodiversity^78,79^. Martin et al.^79^ state that silvicultural alternatives such as continuous cover by using PCs or retention forestry have the potential to restore and protect the habitats and functions of boreal forests. Our results shown that in order to fulfill the current AAC level, the BAU scenario harvested 1.32, 2.04, and 1.80% of the annual managed area in MU1, MU2, and MU3 respectively, while those thresholds could be doubled under S4 and S5. All previously mentioned evidence indicates that PCs could help achieve sustainable management, yet the big challenge will be operational barriers. In fact, in order to harvest the same volume using PC as with CC, PC must be applied in different places and requires greater access, as compared to CC, thereby making PC more costly, as well as resulting in a potential loss of carbon stocks due to the extent of the required road network^6^.

The current goal of forest management in Quebec and in many jurisdictions in the world is to be ecosystem-based, which aims to maintain historical forest composition and structure in an attempt to imitate natural disturbance regimes and preserve natural vegetation patterns^6,80^. Accordingly, our two proposed strategies, S4 and S5, may achieve ecosystem-based management targets since they deviate the least from the vegetation pattern and age structure of the natural scenario. However, the inclusion of more PCs requires increased roads networks with consequent losses in forest area and associated carbon sinks, leading to lower landscape connectivity and increased habitat fragmentation for fauna (*i.e*. caribou)^81^, thus requiring additional effort to consider more ecosystem services in forest landscape models. On the other hand, it seems that using PC based strategies could maintain greater coniferous cover and better preserve current habitats than those based on CC and CPRS. PC also provides quality wood with added value because of higher stem diameters and lower impacts on wildlife at a local scale^82^. In fact, PC maintained mature and old-growth forests, which are distinguished by large-diameter trees, whereas CC increased young forests, which are characterized by lower-diameter trees. However, the implementation of strategies with more PCs requires further road access and appropriate infrastructure, meaning a higher investment cost and increased fragmentation of fauna habitats at the landscape scale^6,14^.

### Modelling limitations and improvements

Our study examines a gradient of forest management strategies running from high to lower harvest intensities at the stand scale and does not account for all of the many site-level conditions. This study does not consider paludified soil in MU1. Although the waterlogging and drought tolerance parameters (H1–H4) were considered, nutrient limitations also reduce productivity, and this is not currently modelled in PnET-Succession. The PnET model simulates stand scale dynamics and extrapolates the results at the landscape scale, hence the integration of the nutrient-stand relationship in the model could be a promising improvement. In addition, the model was optimistic about broadleaved species regeneration under the climate change effect. In fact, the current soil conditions (paludification in MU1, organic and nutrients-poor soils) may limit dispersal for most commercial broadleaved species^83^. In the same MU, Ameray et al^14^ report that under a broadleaved species dispersal restriction scenario, the open forest woodlands will increase for both the RCP4.5 and RCP8.5 scenarios, with more than 10 and 20% in MU1 and MU2, respectively. In contrast, in MU3 the open forest woodland will be around 10% for RCP4.5 and RCP8.5. Consequently, it is possible that some of the increases in projected productivity under the RCP 2.6, 4.5, and 8.5 emissions scenarios may not be realized because of nutrient and soil limitations.

In the boreal forest, the carbon cycle depends on the long-term balance between vegetative carbon inputs from litterfall and root turnover and carbon outputs derived from organic matter decomposition^84^. PCs may reduce decomposition and increase soil carbon storage. In the PC system, higher productivity (i.e., more litterfall input) and lower decomposition rates could be achieved by maintaining a constant level of growing biomass^29^. Future assessments need to integrate the soil carbon pool and estimate both net ecosystem and net biome production, as well as the potential for the forest sector to mitigate climate change, by incorporating harvested wood products in the balance. The PCs positive effect on productivity could be overestimated because our study did not consider the forest edge effect and the interactions with windthrow and SBW. We also highlight that the base harvest model used from LANDIS-II utilizes annual harvested areas rather than volumes, which explains why the model removes less or more biomass than the AAC (Fig. 8). However, our experiment showed the long-term consequences of alternative forest management strategies at the landscape scale compared to the current BAU under climate change effects. Despite current limitations, our study provides a proof-of-concept assessment of the ability of a mechanistic forest landscape model to conduct simulations in order to reduce the uncertainty surrounding the ability of climate-adaptive silvicultural strategies to achieve their stated objectives^85,86^.

## Conclusions

Boreal forests in eastern Canada are undergoing intensive harvesting using CC systems (CC and CPRS), requiring new strategies to achieve sustainability and contribute to climate change mitigation and adaptation. This study indicates that the inclusion of more partial cuts could stabilize forest carbon under the current climate and may even exceed that of a natural scenario (no-harvest) under climate change. Furthermore, in our simulations, PC-based scenarios (S4, S5 and S6) reduced the occupancy of broadleaved species, maintained greater coniferous cover, and allowed a larger contribution of coniferous trees in the future merchantable harvested biomass, mainly after 2110. Under different emission pathways, the losses from CC-based scenarios (S1, S2 and S3) could be compensated in the long term, since it accelerated the expansion of trembling aspen and white birch, while those based on PC-systems increased coniferous cover retention, but that shift in composition could prove challenging to the forest sector. The application of PC-based scenarios under current climate conditions requires forest infrastructural reorganization which will be a big challenge in the future. In addition, the S6 scenario where the entire annual managed area is undergoing PCs treatments might not fulfill industrial needs under the current climate. The application of S4 and S5 requires increasing the coniferous reforestation rate in Eastern regions (MU3) to 8.3–16.7% of the annual harvested area. In addition, CC-based scenarios harvest less area with high annual harvested biomass compared to those based on PC systems, requiring a better coupling of harvested wood products’ life-cycle analyses with landscape dynamics models in future research.

### Supplementary Information

Supplementary Information.

## Data Availability

To ensure the applicability, transparency and future improvement, the datasets and all the input parameters used during the current study are available in GitHub: https://github.com/Ameray/PhD-thesis/tree/main/chapter2.

## References

[1] Dixon RK (1994). Carbon pools and flux of global forest ecosystems. Science.

[2] Pan Y (2011). A large and persistent carbon sink in the world’s forests. Science.

[3] Gauthier S, Bernier P, Kuuluvainen T, Shvidenko AZ, Schepaschenko DG (2015). Boreal forest health and global change. Science.

[4] Landry G (2021). Mitigation potential of ecosystem-based forest management under climate change: A case study in the boreal-temperate forest ecotone. Forests.

[5] Smyth CE (2014). Quantifying the biophysical climate change mitigation potential of Canada’s forest sector. Biogeosciences.

[6] Ameray, A., Bergeron, Y., Valeria, O., Girona, M. & Cavard, X. Forest carbon management: A review of silvicultural practices and management strategies across boreal, tropical, and temperate forests. *Curr. For. Rep.* (2021).

[7] Bergeron Y, Gauthier S, Flannigan M, Kafka V (2004). Fire regimes at the transition between Mixedwood and coniferous boreal forest in Northwestern Quebec. Ecology.

[8] Bergeron Y (2006). Past, current, and future fire frequencies in Quebec’s commercial forests: Implications for the cumulative effects of harvesting and fire on age-class structure and natural disturbance-based management. Can. J. For. Res..

[9] Bergeron Y, Gauthier S, Kafka V, Lefort P, Lesieur D (2001). Natural fire frequency for the eastern Canadian boreal forest: consequences for sustainable forestry. Can. J. For. Res..

[10] Navarro L, Morin H, Bergeron Y, Girona MM (2018). Changes in spatiotemporal patterns of 20th century spruce budworm outbreaks in eastern Canadian boreal forests. Front. Plant Sci..

[11] Bouchard M, Pothier D, Ruel J-C (2009). Stand-replacing windthrow in the boreal forests of eastern Quebec. Can. J. For. Res..

[12] Boulanger Y, Gauthier S, Burton PJ (2014). A refinement of models projecting future Canadian fire regimes using homogeneous fire regime zones. Can. J. For. Res..

[13] Augustin F (2022). Projected changes in fire activity and severity feedback in the spruce—feather moss forest of western Quebec, Canada. Trees For. People.

[14] Ameray A, Bergeron Y, Cavard X (2023). Climate change may increase Quebec boreal forest productivity in high latitudes by shifting its current composition. Front. For. Glob. Chang..

[15] Boulanger Y (2012). Dendrochronological reconstruction of spruce budworm (*Choristoneura fumiferana*) outbreaks in southern Quebec for the last 400 years. Can. J. For. Res..

[16] Liu Z (2019). Simulation and analysis of the effect of a spruce budworm outbreak on carbon dynamics in boreal forests of Quebec. Ecosystems.

[17] Dymond C (2010). Future spruce budworm outbreak may create a carbon source in eastern Canadian forests. Ecosystems.

[18] Mayer M, Sandén H, Rewald B, Godbold DL, Katzensteiner K (2017). Increase in heterotrophic soil respiration by temperature drives decline in soil organic carbon stocks after forest windthrow in a mountainous ecosystem. Funct. Ecol..

[19] Mitchell SJ (2012). Wind as a natural disturbance agent in forests: A synthesis. For. An Int. J. For. Res..

[20] Don A (2012). No rapid soil carbon loss after a windthrow event in the High Tatra. For. Ecol. Manage..

[21] Kurz WA (2009). CBM-CFS3: A model of carbon-dynamics in forestry and land-use change implementing IPCC standards. Ecol. Modell..

[22] Schuur EAG (2008). Vulnerability of permafrost carbon to climate change: Implications for the global carbon cycle. Bioscience.

[23] Gustafson EJ, Miranda B, Sturtevant B (2018). Can future CO_2_ concentrations mitigate the negative effects of high temperature and longer droughts on forest growth?. Forests.

[24] IPCC. *Climate Change: The 5th Assessment Report of the Intergovernmental Panel on Climate Change*. (2014).

[25] Peng (2011). A drought-induced pervasive increase in tree mortality across Canada’s boreal forests. Nat. Clim. Chang..

[26] Zhuo W, Dai E, Wu Z, Lin M (2020). Assessing differences in the response of forest aboveground biomass and composition under climate change in subtropical forest transition zone. Sci. Total Environ..

[27] Gustafson EJ (2015). Integrating ecophysiology and forest landscape models to improve projections of drought effects under climate change. Glob. Chang. Biol..

[28] De Bruijn A (2014). Toward more robust projections of forest landscape dynamics under novel environmental conditions: embedding PnET within LANDIS-II. Ecol. Modell..

[29] Taylor AR, Wang JR, Kurz WA (2008). Effects of harvesting intensity on carbon stocks in eastern Canadian red spruce (*Picea rubens*) forests: An exploratory analysis using the CBM-CFS3 simulation model. For. Ecol. Manage..

[30] Tian H (2015). Global patterns and controls of soil organic carbon dynamics as simulated by multiple terrestrial biosphere models: Current status and future directions. Global Biogeochem. Cycles.

[31] Liu S (2011). Simulating the impacts of disturbances on forest carbon cycling in North America: Processes, data, models, and challenges. J. Geophys. Res. Biogeosci..

[32] Goulden ML (2011). Patterns of NPP, GPP, respiration, and NEP during boreal forest succession. Glob. Change Biol..

[33] Lee J, Morrison IK, Leblanc J-D, Dumas MT, Cameron DA (2002). Carbon sequestration in trees and regrowth vegetation as affected by clearcut and partial cut harvesting in a second-growth boreal Mixedwood. For. Ecol. Manage..

[34] Noormets A (2015). Effects of forest management on productivity and carbon sequestration: A review and hypothesis. For. Ecol. Manage..

[35] Pamerleau-Couture É, Krause C, Pothier D, Weiskittel A (2015). Effect of three partial cutting practices on stand structure and growth of residual black spruce trees in north-eastern Quebec. For. An Int. J. For. Res..

[36] MRNF. Forêt ouverte: inventaire forestier national. https://www.foretouverte.gouv.qc.ca/?context=_catalogue_complet&zoom=6&center=-73,51&invisiblelayers=*&visiblelayers=pee_index_pdf_mai2015,fond&llcv=1 (2010).

[37] Peng C, Liu J, Dang Q, Apps MJ, Jiang H (2002). TRIPLEX: A generic hybrid model for predicting forest growth and carbon and nitrogen dynamics. Ecol. Modell..

[38] Jobidon R, Bergeron Y (2015). Assessing the biophysical potential for sustainable forest management: a case study from Quebec’s boreal forest/Evaluation du potentiel biophysique pour un amenagement durable des forets: le cas de la foret boreale du Quebec. Can. J. For. Res..

[39] Wang T, Hamann A, Spittlehouse D, Carroll C (2016). Locally downscaled and spatially customizable climate data for historical and future periods for North America. PLoS ONE.

[40] Rogelj J (2016). Paris Agreement climate proposals need a boost to keep warming well below 2 °C. Nature.

[41] Collins, M. *et al.* Long-term climate change: projections, commitments and irreversibility. In *Climate Change 2013—The Physical Science Basis: Contribution of Working group I to the 5th Assessment Report of the Intergovernmental Panel on Climate Change* 1029–1136 (Cambridge University Press, 2013).

[42] Gustafson, E. & Miranda, B. PnET-Succession - LANDIS-II. 74 http://www.landis-ii.org/extensions/pnet-succession (2019).

[43] Aber JD, Federer CA (1992). A generalized, lumped-parameter model of photosynthesis, evapotranspiration and net primary production in temperate and boreal forest ecosystems. Oecologia.

[44] Gustafson EJ, Kern CC, Kabrick JM (2023). Can assisted tree migration today sustain forest ecosystem goods and services for the future?. For. Ecol. Manage..

[45] Pothier, D. & Savard, F. Actualisation des tables de production pour les principales espèces du Québec. *Gouv. du Québec, ministère des Ressources Nat. Bibliothèque Natl. du Québec. RN98–3054* (1998).

[46] Boulanger Y (2017). Climate change impacts on forest landscapes along the Canadian southern boreal forest transition zone. Landsc. Ecol..

[47] Duchesne L, Ouimet R (2021). Digital mapping of soil texture in ecoforest polygons in Quebec. Canada. PeerJ.

[48] Scheller, R. M. & Domingo, J. B. LANDIS-II Base Fire v4.0 Extension User Guide. *Reproduction* 0–9 (2018).

[49] Scheller, R. M. *et al.* LANDIS-II Base Wind v3.0 Extension User Guide. 1–11 (2018).

[50] Scheller, R. M., Sturtevant, B. R., Gustafson, E. J., Miranda, B. R. & Zollner, P. A. Biomass Harvest v4.3 LANDIS-II Extension User Guide. 0–9 (2019).

[51] Sturtevant, B. R., He, H. S., Scheller, R. M. & Miranda, B. R. LANDIS-II Biological Disturbance Agent v2 . 0 Extension User Guide. 0–19 (2019).

[52] Molina E (2021). Projecting future aboveground biomass and productivity of managed eastern Canadian mixedwood boreal forest in response to climate change. For. Ecol. Manage..

[53] Tremblay JA (2018). Harvesting interacts with climate change to affect future habitat quality of a focal species in eastern Canada’s boreal forest. PLoS ONE.

[54] Boulanger Y (2019). Climate change will affect the ability of forest management to reduce gaps between current and presettlement forest composition in southeastern Canada. Landsc. Ecol..

[55] Forestier en chef. Forestier en chef—Possibilités forestières 2023–2028. https://forestierenchef.gouv.qc.ca/possibilites-forestieres/ (2022).

[56] Krofcheck DJ, Remy CC, Keyser AR, Hurteau MD (2019). Optimizing forest management stabilizes carbon under projected climate and wildfires. J. Geophys. Res. Biogeosci..

[57] D’Orangeville L (2018). Beneficial effects of climate warming on boreal tree growth may be transitory. Nat. Commun..

[58] Beck PSA (2011). Changes in forest productivity across Alaska consistent with biome shift. Ecol. Lett..

[59] Dhital N (2015). Adaptation potential of ecosystem-based management to climate change in the eastern Canadian boreal forest. J. Environ. Plan. Manag..

[60] Cavard X, Bergeron Y, Chen HYH, Pare D (2010). Mixed-species effect on tree aboveground carbon pools in the east-central boreal forests. Can. J. For. Res..

[61] Cavard X (2011). Competition and facilitation between tree species change with stand development. Oikos.

[62] Stuenzi SM, Schaepman-Strub G (2020). Vegetation trajectories and shortwave radiative forcing following boreal forest disturbance in eastern Siberia. J. Geophys. Res. Biogeosci..

[63] Paradis L, Thiffault E, Achim A (2019). Comparison of carbon balance and climate change mitigation potential of forest management strategies in the boreal forest of Quebec (Canada). For. An Int. J. For. Res..

[64] Boulanger Y, Puigdevall PJ (2021). Boreal forests will be more severely affected by projected anthropogenic climate forcing than Mixedwood and northern hardwood forests in eastern Canada. Landsc. Ecol..

[65] Peng C, Jiang H, Apps MJ, Zhang Y (2002). Effects of harvesting regimes on carbon and nitrogen dynamics of boreal forests in central Canada: A process model simulation. Ecol. Modell..

[66] Walker XJ, Mack MC, Johnstone JF (2017). Predicting ecosystem resilience to fire from tree ring analysis in black spruce forests. Ecosystems.

[67] Paixao C, Krause C, Morin H, Achim A (2019). Wood quality of black spruce and balsam fir trees defoliated by spruce budworm: A case study in the boreal forest of Quebec, Canada. For. Ecol. Manage..

[68] Bergeron Y (2017). Projections of future forest age class structure under the influence of fire and harvesting: Implications for forest management in the boreal forest of eastern Canada. For. An Int. J. For. Res..

[69] Splawinski TB, Cyr D, Gauthier S, Jetté J-P, Bergeron Y (2019). Analyzing risk of regeneration failure in the managed boreal forest of northwestern Quebec. Can. J. For. Res..

[70] Kurz WA (2013). Carbon in Canada’s boreal forest—A synthesis. Environ. Rev..

[71] Dulamsuren C (2021). Organic carbon stock losses by disturbance: Comparing broadleaved pioneer and late-successional conifer forests in Mongolia’s boreal forest. For. Ecol. Manage..

[72] Guignabert A (2020). Combining partial cutting and direct seeding to overcome regeneration failures in dune forests. For. Ecol. Manage..

[73] Simard WS (2020). Harvest intensity effects on carbon stocks and biodiversity are dependent on regional climate in Douglas-fir forests of British Columbia. Front. Forests Glob. Change.

[74] Montoro Girona M, Morin H, Lussier J-M, Ruel J-C (2019). Post-cutting mortality following experimental silvicultural treatments in unmanaged boreal forest stands. Front. For. Glob. Chang..

[75] Boucher J-F, Tremblay P, Gaboury S, Villeneuve C (2012). Can boreal afforestation help offset incompressible GHG emissions from Canadian industries?. Process Saf. Environ. Prot..

[76] Gaboury S, Boucher J-F, Villeneuve C, Lord D, Gagnon R (2009). Estimating the net carbon balance of boreal open woodland afforestation: A case-study in Québec’s closed-crown boreal forest. For. Ecol. Manage..

[77] FAO. *Global Forest Resources Assessment 2020 Main report*. *Forestry Chronicle* vol. 16 https://www.fao.org/documents/card/en/c/ca9825en (2020).

[78] Harvey BD, Leduc A, Gauthier S, Bergeron Y (2002). Stand-landscape integration in natural disturbance-based management of the southern boreal forest. For. Ecol. Manage..

[79] Martin M (2022). Irregular forest structures originating after fire: An opportunity to promote alternatives to even-aged management in boreal forests. J. Appl. Ecol..

[80] Bergeron Y, Harvey B, Leduc A, Gauthier S (1999). Forest management guidelines based on natural disturbance dynamics: Stand-and forest-level considerations. For. Chron..

[81] Prima M-C (2019). A landscape experiment of spatial network robustness and space-use reorganization following habitat fragmentation. Funct. Ecol..

[82] St-Laurent M-H (2022). Lowering the rate of timber harvesting to mitigate impacts of climate change on boreal caribou habitat quality in eastern Canada. Sci. Total Environ..

[83] Gewehr S, Drobyshev I, Berninger F, Bergeron Y (2014). Soil characteristics mediate the distribution and response of boreal trees to climatic variability. Can. J. For. Res..

[84] Laganière J (2013). Stability of soil carbon stocks varies with forest composition in the Canadian boreal biome. Ecosystems.

[85] Gustafson EJ, Miranda BR, Dreaden TJ, Pinchot CC, Jacobs DF (2022). Beyond blight: Phytophthora root rot under climate change limits populations of reintroduced American chestnut. Ecosphere.

[86] Gustafson EJ (2020). Climate adaptive silviculture strategies: How do they impact growth, yield, diversity and value in forested landscapes?. For. Ecol. Manage..

