# Supplementary material for: Modelling the potential of forest management to mitigate climate change in Eastern Canadian forests
Source: Sci Rep. 2023 Sep 4;13:14506. doi: 10.1038/s41598-023-41790-2 (PMC10477314; doi:10.1038/s41598-023-41790-2)

Supplementary materials 1. The life-history attributes for the 8 species according to Boulanger et *al.* (2017) (L: longevity (years), SM: sexual maturity, ST: shade tolerance, SDD: seeds dispersal distance, VRP: vegetation reproduction probability, PFR: post-fire regeneration)

| Species | L | SM | **ST** | **FT** | **SDD** | | **VRP** | **VRP min age** | **VRP**  **max age** | **PFR** |
| --- | --- | --- | --- | --- | --- | --- | --- | --- | --- | --- |
|  |  |  |  |  | **effective** | **Max** |  |  |  |  |
| *Abies balsamea* | 150 | 30 | 5 | 1 | 25 | 160 | 0 | 0 | 0 | none |
| *Betula alleghaniensis* | 220 | 40 | 3 | 1 | 100 | 400 | 0.1 | 10 | 180 | resprout |
| *Betula papyrifera* | 140 | 20 | 2 | 1 | 100 | 1000 | 0.5 | 10 | 70 | resprout |
| *Larix laricina* | 160 | 40 | 1 | 1 | 50 | 200 | 0 | 0 | 0 | none |
| *Picea glauca* | 200 | 30 | 3 | 2 | 100 | 300 | 0 | 0 | 0 | none |
| *Picea marian* | 220 | 30 | 4 | 2 | 80 | 200 | 0 | 0 | 0 | serotiny |
| *Pinus banksiana* | 140 | 20 | 1 | 2 | 30 | 100 | 0 | 0 | 0 | serotiny |
| *Populus tremuloides* | 130 | 20 | 1 | 2 | 500 | 5000 | 0.9 | 10 | 130 | resprout |

Supplementary material 2. The total area of each management unit (three MUs: North-of-Quebec (MU1), Saguenay-Lac-Saint-Jean (MU2), and Côte-Nord (MU3)), with allowable annual cut (AAC) determined for the current planning cycle (2023-2028). Annual harvested area (%) per management scenario to fulfill the AAC.

| **MU** | **Total area (Mha)** | **AAC (Mg)** | **Annual managed area (%) per scenario** | | | | | |
| --- | --- | --- | --- | --- | --- | --- | --- | --- |
|  |  |  | **S1** | **S2** | **S3** | **S4** | **S5** | **S6** |
| **MU1** | 0.62 | 245808 | 1.18 | 1.32 | 1.48 | 1.70 | 2.21 | 2.89 |
| **MU2** | 1.16 | 511644 | 1.95 | 2.04 | 2.20 | 2.56 | 2.72 | 4.27 |
| **MU3** | 1.14 | 405547 | 1.70 | 1.80 | 1.83 | 2.07 | 2.55 | 3.55 |

**Supplementary material 3.** Used treatment at stand scale and % of managed area per treatment for the BAU (historic of 1970-2010) per MU and their management areas. CC + reforestation reflects that clear-cut or CPRS is followed by replanting, due to a lower soil seed banks and regeneration rate.

| **MU** | **Management area** | **Map**  **code** | **area (10^3^)** | **Used treatment at stand scale and % of managed area per treatment under BAU** | | | | | |
| --- | --- | --- | --- | --- | --- | --- | --- | --- | --- |
|  |  |  |  | **CC+**  **regeneration** | **CC+**  **reforestation** | **CPRS95** | **PC75** | **PC50** | **PC25** |
| 1 | Macamic lake | 1 | 31.7 | 40.91 | 9.40 | 44.80 | 0.88 | 0.00 | 4.02 |
|  | Obalski lake | 2 | 82.3 | 73.70 | 17.82 | 6.39 | 0.18 | 0.00 | 1.91 |
|  | Mistaouac lake | 3 | 225.2 | 39.74 | 23.55 | 33.07 | 1.97 | 0.28 | 1.39 |
|  | Grasset lake | 4 | 279.2 | 34.16 | 8.49 | 56.37 | 0.10 | 0.02 | 0.86 |
|  | Weighted average | | | 41.80 | 15.26 | 40.64 | 0.83 | 0.11 | 1.36 |
| 2 | Connelly lake | 1 | 80.0 | 28.03 | 5.61 | 58.17 | 0.32 | 6.39 | 1.48 |
|  | Onatchiway lake | 2 | 72.1 | 40.31 | 4.77 | 47.08 | 0.00 | 6.34 | 1.49 |
|  | Pipmuacan Reservoir | 3 | 300.3 | 37.28 | 17.06 | 44.30 | 0.16 | 0.94 | 0.25 |
|  | Péribonka lake | 4 | 465.8 | 10.35 | 12.96 | 72.46 | 3.72 | 0.52 | 0.00 |
|  | Hirondelles lake | 5 | 232.3 | 0.89 | 0.72 | 92.78 | 4.62 | 1.00 | 0.00 |
|  | Weighted average | | | 18.57 | 10.54 | 66.63 | 2.50 | 1.50 | 0.26 |
| 3 | Dionne lake | 1 | 149.9 | 86.30 | 0.07 | 11.74 | 0.00 | 1.60 | 0.30 |
|  | LM Forestville Lake | 2 | 222.7 | 93.40 | 1.82 | 2.61 | 0.01 | 2.14 | 0.01 |
|  | CBM lake | 3 | 213.7 | 0.01 | 1.42 | 97.31 | 0.00 | 1.26 | 0.00 |
|  | Manic Reservoir | 4 | 562.1 | 47.12 | 2.22 | 47.07 | 0.54 | 3.03 | 0.02 |
|  | Weighted average | | | 52.44 | 1.71 | 43.19 | 0.27 | 2.34 | 0.05 |

Supplementary materials 4. The difference (*Δ*; %) of composition percentage between natural scenario and management scenarios in the three MU between 2010 (year 0) and 2310 (year 300) under RCP2.6 and RCP4.5 climate change scenario. (BSPF: black spruce pure forests, OCPF: Other coniferous pure forests, BPF: broadleaves pure forests, BSJP: black spruce and jack pine, BSAB: black spruce and balsam fir, BSOC: black spruce and other coniferous, OCMF: Other coniferous mixed forests, BMF: broadleaves mixed forests; BsBMF: black spruce and broadleaves mixed forests, OCBMF: Other coniferous and broadleaves mixed forests).


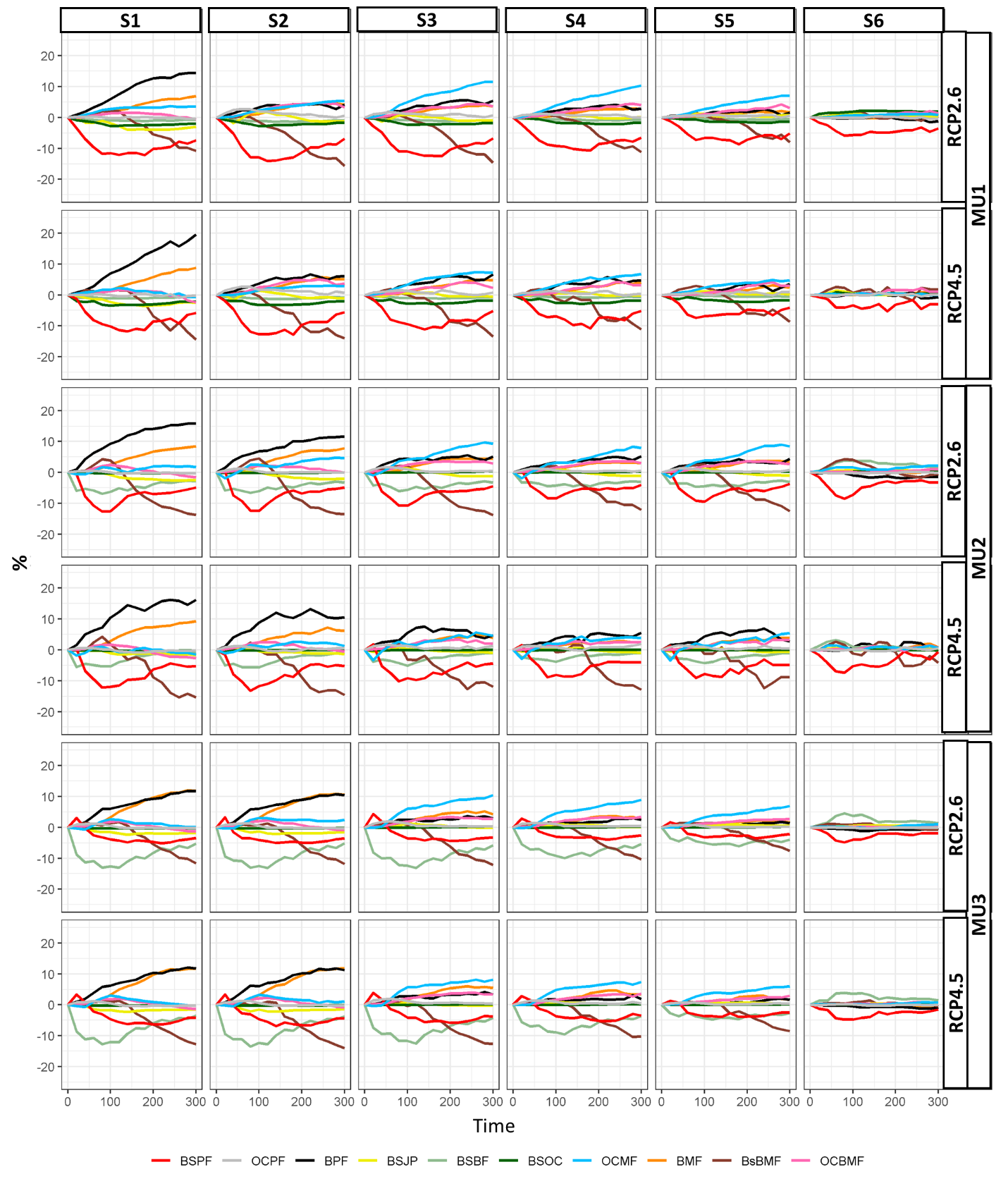


Supplementary materials 5. The occupancy area (%) of forest type in the three MU between 2010 (year 0) and 2310 (year 300) under climate change scenarios and no-harvest scenario, where the natural disturbances (SBW, wind, fire) were considered (BSPF: black spruce pure forests, OCPF: Other coniferous pure forests, BPF: broadleaves pure forests, BSJP: black spruce and jack pine, BSBF: black spruce and balsam fir, BSOC: black spruce and other coniferous, OCMF: Other coniferous mixed forests, BMF: broadleaves mixed forests; BsBMF: black spruce and broadleaves mixed forests, OCBMF: Other coniferous and broadleaves mixed forests; Em: empty land which represents open forest woodland).


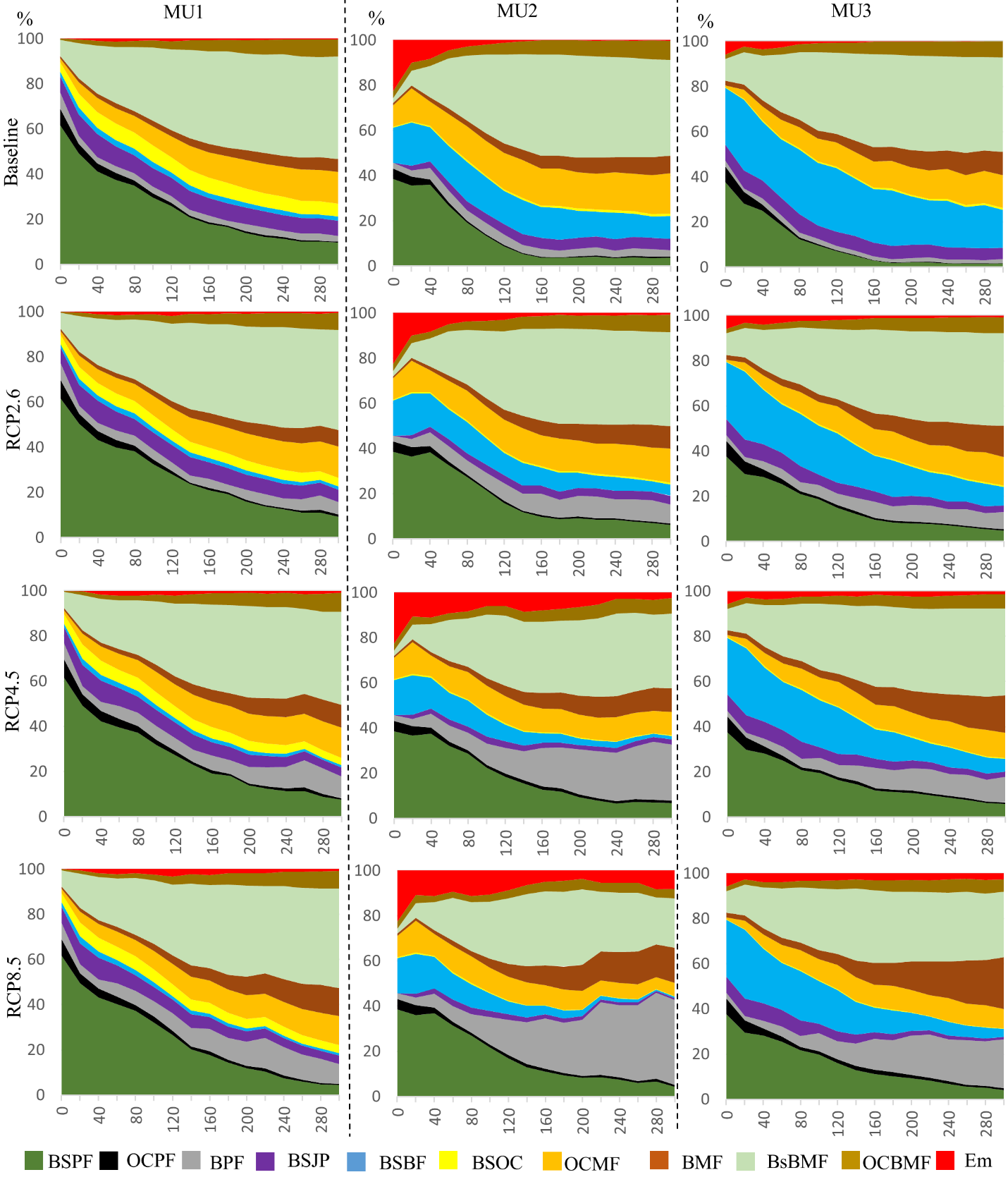


Supplementary materials 6. Age structure (young forest ≤40; 40<mature forest≤100; 100< old growth forest) under current climate and RCP8.5 scenarios, for natural evolution (S0; no-harvest), BAU (S2) and PC-based strategies (S4, S5, S6).


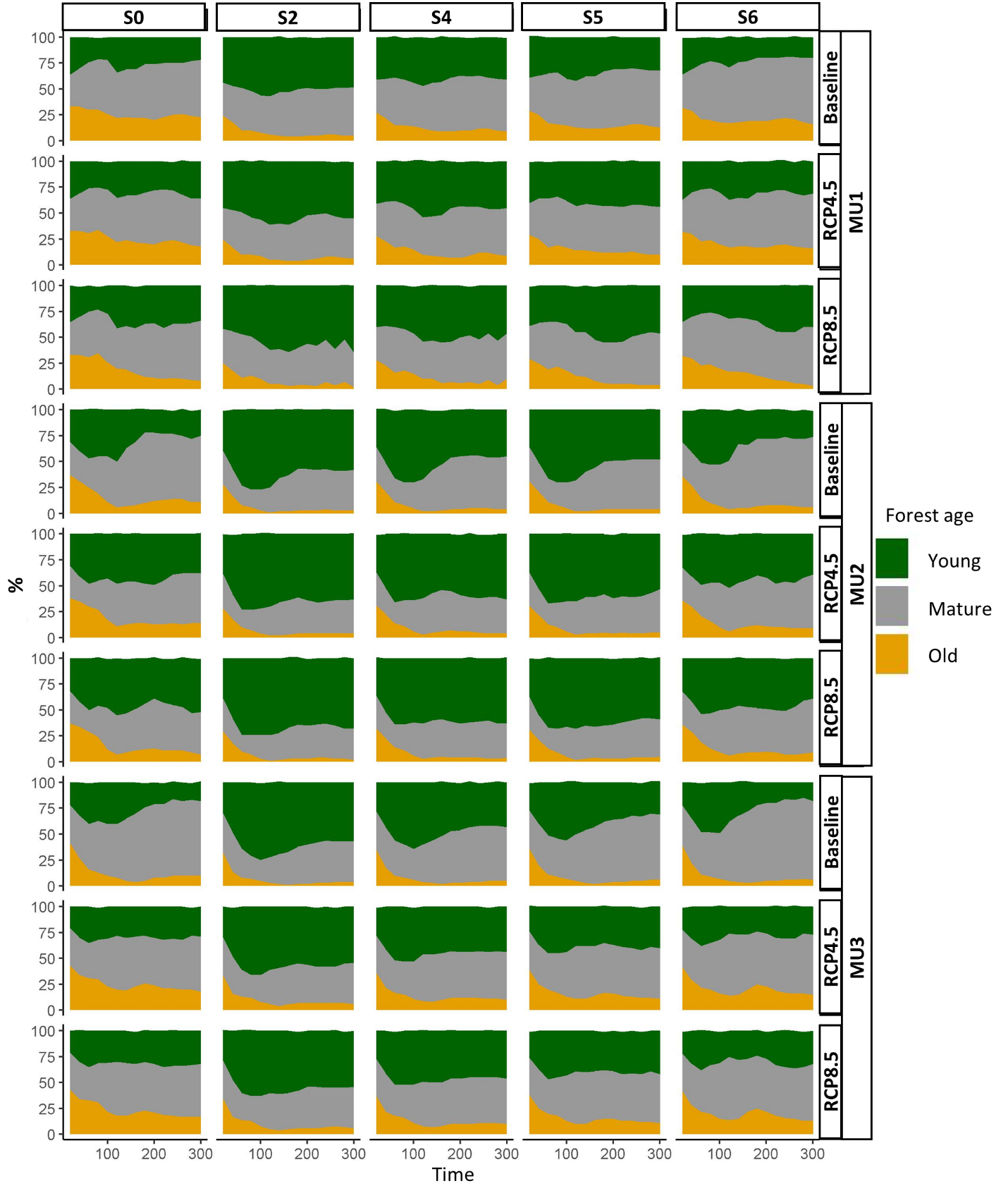


Supplementary materials 7. Carbon losses (-) or gain (+) (tC ha-1 yr-1) average projection coniferous and broadleaves species in the three management units (1, 2, 3) under different natural disturbances (Winds, fires, SBW) and climate change scenarios. The averages were estimated for the entire study period (2010-2310) and the model was run by 20 years time step.


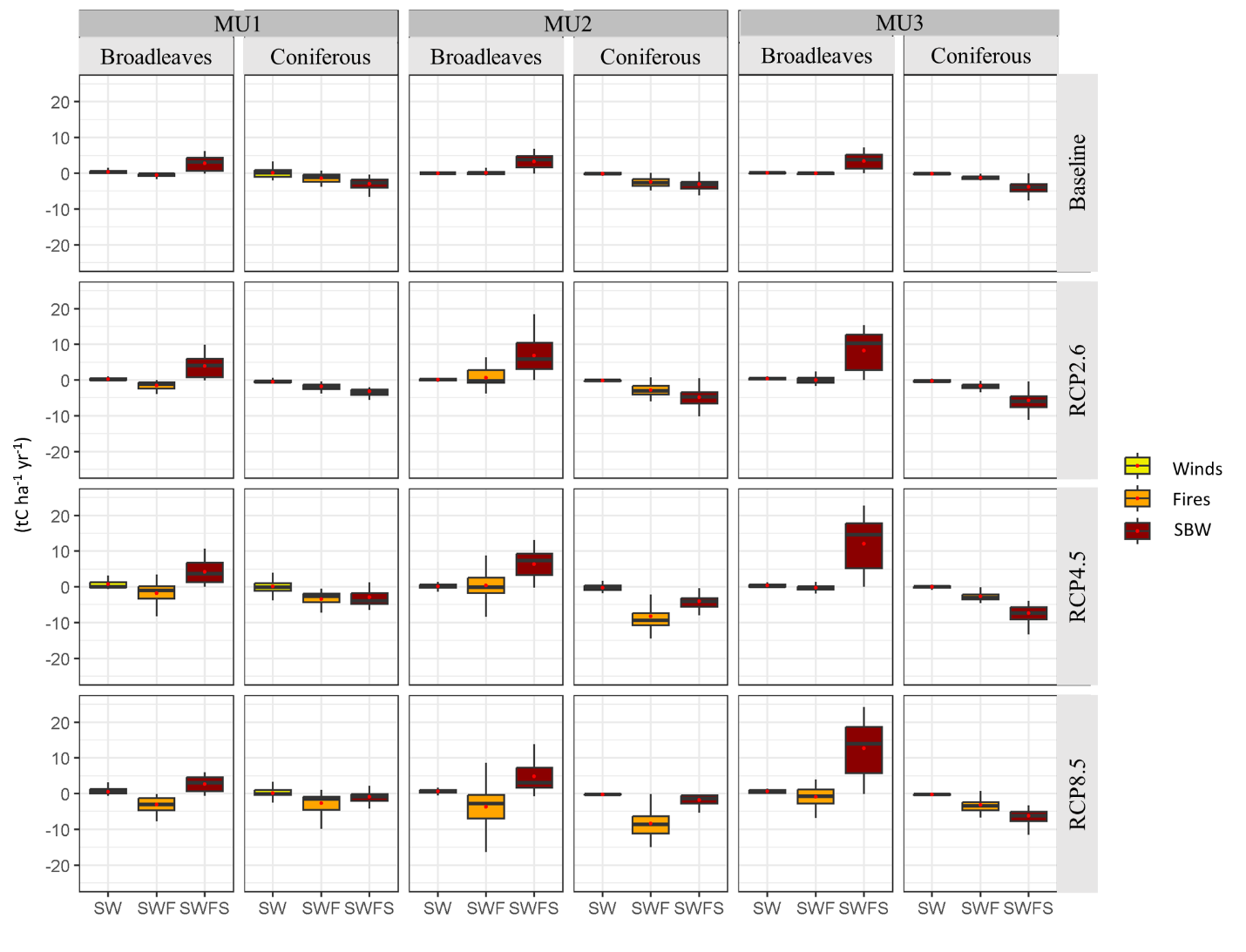


Supplementary materials 8 a). The relative changes in biomass carbon storage compared to the natural scenario (S0) expressed in percentage.


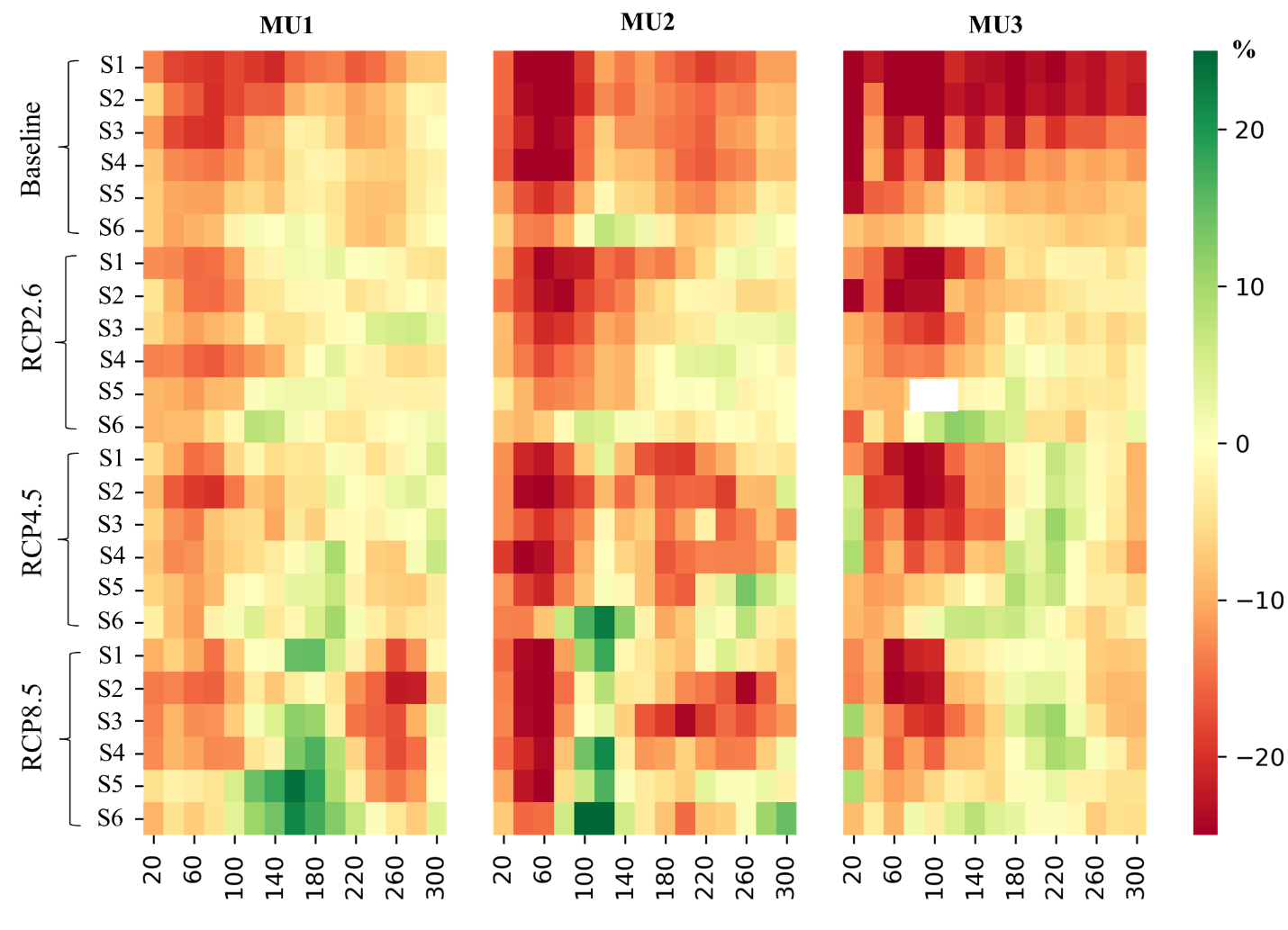


Supplementary materials 8 b). The living biomass carbon storage differences (Δi,j) from 2010 (year 0) to 2310 (year 300) between management scenarios and no harvest scenario (S0: natural disturbances only) with their confident interval.


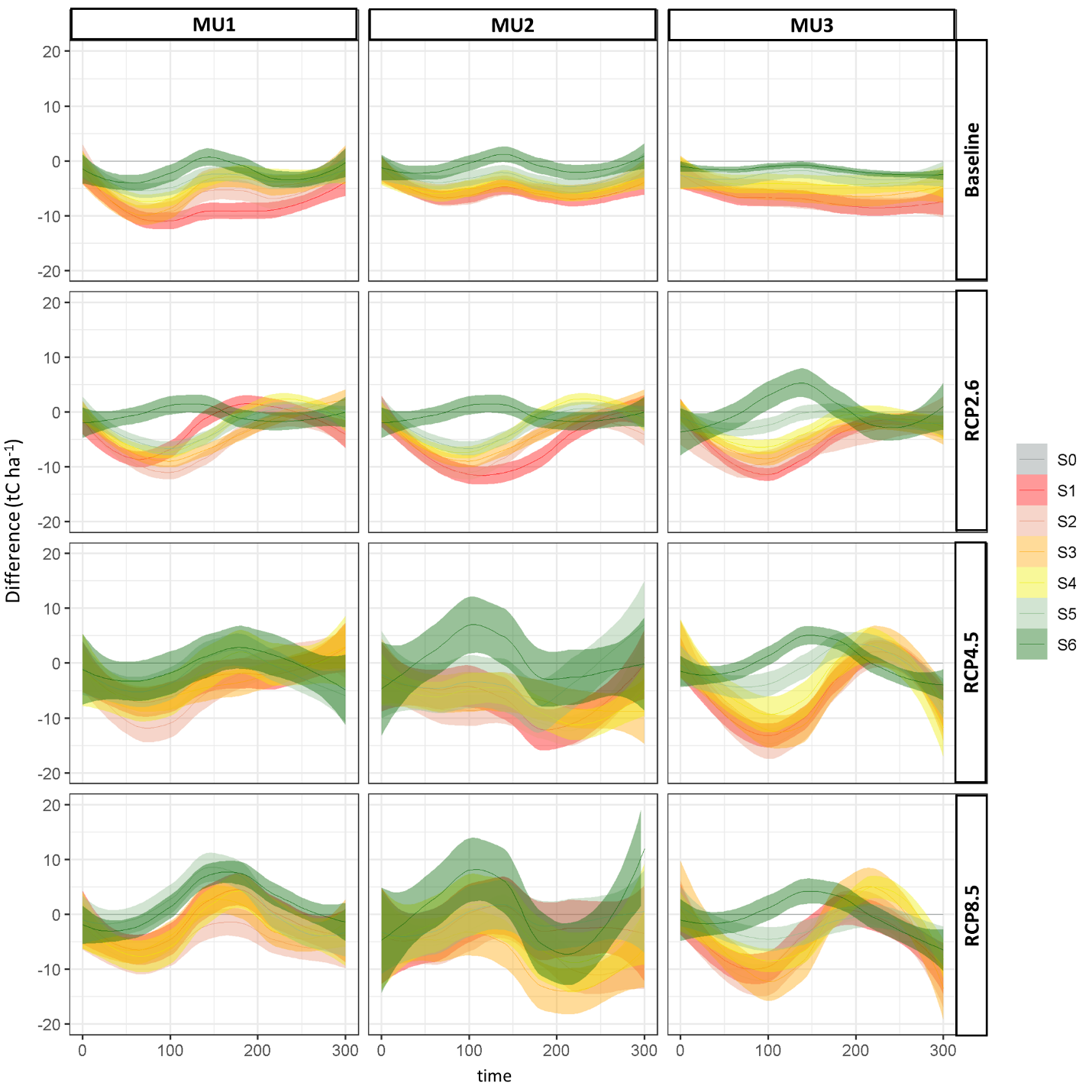


Supplementary materials 9. The relative percentage of ACC per species in the three management units under climate change scenarios and six management strategies from 2010 (year 0) to 2310 (year 300) (legend: BS: black spruce, WS: white spruce, JP: jack pine, BF: balsam fir, LT: larch tree, WB: white birch, TA: trembling aspen). This SM reflects the contribution of each species in annual harvested biomass.


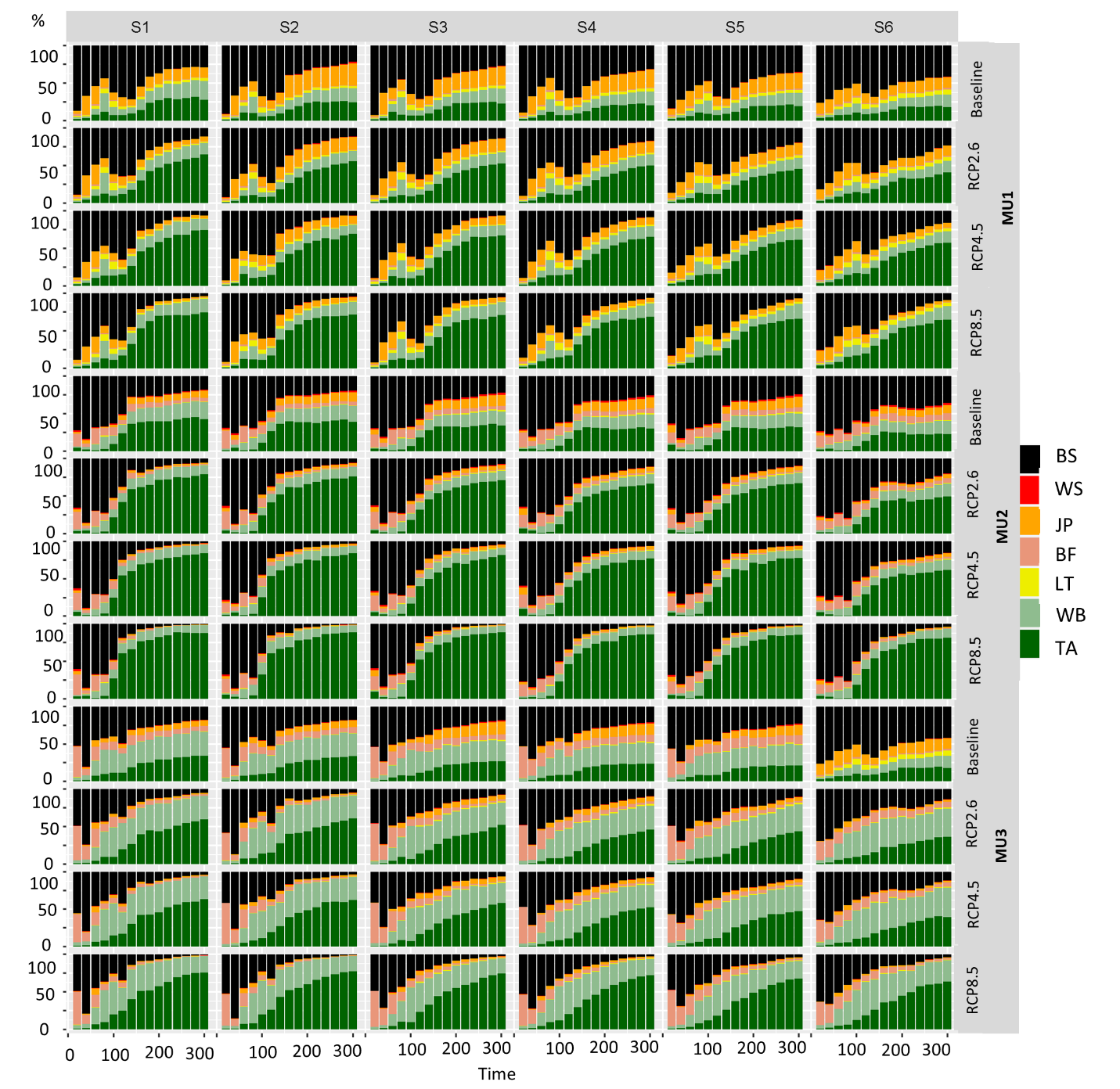


Supplementary material 10. Above-ground biomass (AGB) response to silvicultural treatment at stand scale of the black spruce pure forest. PCs stabilized biomass carbon pool compared to CC and CPRS in the long term.


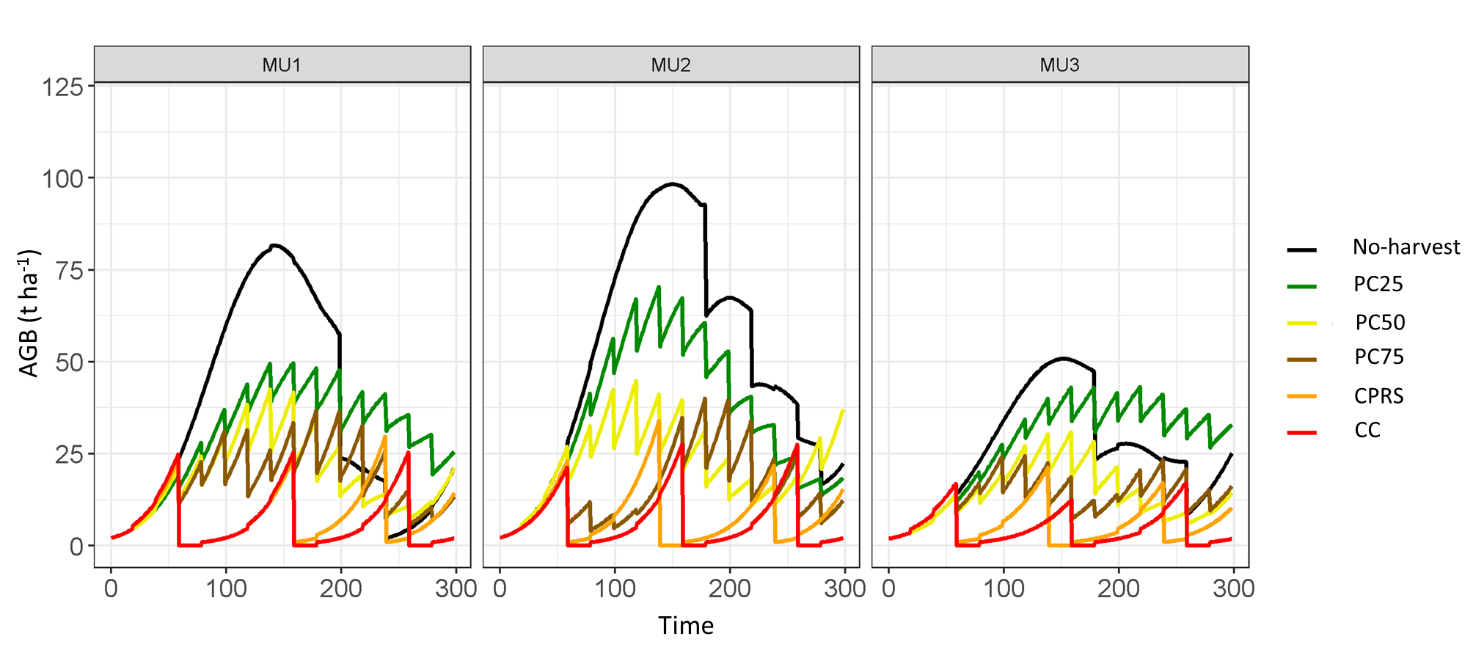

Supplement: Supplementary file 1 — Supplementary Information. [file 41598_2023_41790_MOESM1_ESM.docx]
